# Fanconi anemia and homologous recombination gene variants are associated with functional DNA repair defects *in vitro* and poor outcome in patients with advanced head and neck squamous cell carcinoma

**DOI:** 10.18632/oncotarget.24797

**Published:** 2018-04-06

**Authors:** Caroline V.M. Verhagen, David M. Vossen, Kerstin Borgmann, Floor Hageman, Reidar Grénman, Manon Verwijs-Janssen, Lisanne Mout, Roel J.C. Kluin, Marja Nieuwland, Tesa M. Severson, Arno Velds, Ron Kerkhoven, Mark J. O’Connor, Martijn van der Heijden, Marie-Louise van Velthuysen, Marcel Verheij, Volkert B. Wreesmann, Lodewyk F.A. Wessels, Michiel W.M. van den Brekel, Conchita Vens

**Affiliations:** ^1^ Division of Cell Biology, The Netherlands Cancer Institute, Amsterdam, The Netherlands; ^2^ Department of Head and Neck Oncology and Surgery, The Netherlands Cancer Institute, Amsterdam, The Netherlands; ^3^ Laboratory of Radiobiology and Experimental Radiation Oncology, University Medical Centre Hamburg-Eppendorf, Hamburg, Germany; ^4^ Department of Otorhinolaryngology, Turku University Hospital, University of Turku, Turku, Finland; ^5^ Genomics Core Facility, The Netherlands Cancer Institute, Amsterdam, The Netherlands; ^6^ Division of Molecular Pathology, The Netherlands Cancer Institute, Amsterdam, The Netherlands; ^7^ Oncology Innovative Medicines, AstraZeneca, Saffron Walden, UK; ^8^ Department of Pathology, The Netherlands Cancer Institute, Amsterdam, The Netherlands; ^9^ Department of Radiation Oncology, The Netherlands Cancer Institute, Amsterdam, The Netherlands; ^10^ Molecular Carcinogenesis, The Netherlands Cancer Institute, Amsterdam, The Netherlands; ^11^ Department of Oral and Maxillofacial Surgery, Academic Medical Center, University of Amsterdam, Amsterdam, The Netherlands

**Keywords:** HNSCC, gene variants, homologous recombination, Fanconi anemia, DNA repair

## Abstract

Mutations in Fanconi Anemia or Homologous Recombination (FA/HR) genes can cause DNA repair defects and could therefore impact cancer treatment response and patient outcome. Their functional impact and clinical relevance in head and neck squamous cell carcinoma (HNSCC) is unknown. We therefore questioned whether functional FA/HR defects occurred in HNSCC and whether they are associated with FA/HR variants. We assayed a panel of 29 patient-derived HNSCC cell lines and found that a considerable fraction is hypersensitive to the crosslinker Mitomycin C and PARP inhibitors, a functional measure of FA/HR defects. DNA sequencing showed that these hypersensitivities are associated with the presence of bi-allelic rare germline and somatic FA/HR gene variants. We next questioned whether such variants are associated with prognosis and treatment response in HNSCC patients. DNA sequencing of 77 advanced stage HNSCC tumors revealed a 19% incidence of such variants. Importantly, these variants were associated with a poor prognosis (*p* = 0.027; HR = 2.6, 1.1–6.0) but favorable response to high cumulative cisplatin dose. We show how an integrated *in vitro* functional repair and genomic analysis can improve the prognostic value of genetic biomarkers. We conclude that repair defects are marked and frequent in HNSCC and are associated with clinical outcome.

## INTRODUCTION

Chromosomal stability is governed by DNA damage response and repair processes such as the homologous recombination (HR) and Fanconi Anemia (FA) pathways. The FA-pathway is essential for the repair of DNA interstrand crosslinks and together with elements of the homologous recombination (HR) repair pathway they also strongly determine cellular survival upon exposure to crosslinking agents [1]. Aberrations in the FA/HR-pathway have been reported in multiple cancer types and their therapeutic exploitation has been described [2, 3]. The breast cancer susceptibility genes *BRCA1* and *BRCA2* (*BRCA1/2*), well-known members of the FA/HR-pathway, have a well-described role in hereditary breast and ovarian cancer. Recent DNA sequencing studies highlight the high occurrence of DNA repair gene aberrations; however, the assessment of a functional impact lags behind and their clinical relevance remains poorly defined. Notably, the underlying DNA repair defects in *BRCA1/2* mutated breast and ovarian tumors can be exploited with PARP inhibitors [4–6] further stressing the importance of functional DNA repair defect studies.

Fanconi anemia patients suffer from a condition caused by germline mutations in the Fanconi anemia (FA) genes and have an increased susceptibility to cancer. Head and neck squamous cell carcinoma (HNSCC) is the most common solid cancer in these patients, with a 700-fold increased risk [7, 8]. Sporadic HNSCC is the sixth most common cancer worldwide and its incidence is strongly associated with alcohol consumption, smoking and HPV infection [9, 10]. A considerable proportion of patients is diagnosed at an advanced stage, at which patients are often treated with surgery or a combination of radiotherapy and cisplatin. This combination is effective, although not all patients benefit and less than half of the patients will be cured [11]. In addition, many suffer severe side effects without possibly benefiting from the treatment. New treatment decision aids and alternative therapeutic approaches are therefore urgently needed [12–14].

The strong impact of smoking and alcohol in the development of HNSCC, both likely based on the DNA crosslinking nature of these mutagens [15, 16], suggests a protective role of the FA/HR repair pathway. Meta-analysis has shown the benefit of the addition of crosslinking agents to radiotherapy to improve outcome in HNSCC [11] and may further indicate tumor DNA repair defects to be involved in crosslinker sensitivity. Together these data point to a role of crosslink repair defects, particularly those of the FA/HR pathway, in the etiology and treatment of HNSCC.

In sporadic HNSCC, downregulation of FA gene expression [17] and frequent *FANCF* silencing by methylation was found [18]. Furthermore, copy number alterations [19] and somatic mutations of individual FA genes have been described in HNSCC [20, 21]. A recent study found FA gene variants in HNSCC cell lines that were responsive to a chromosomal breakage assay [22]. Comprehensive genomic analysis of the FA/HR pathway are rare and it is unknown whether these alterations compromise cellular crosslink repair activity, as functional analyses are lacking [23]. Importantly however, the clinical relevance of functional or genetic FA/HR tumor defects has not been elucidated.

In this study we therefore investigate the incidence and properties of functional DNA repair defects in HNSCC by applying multiple functional assays to a large HNSCC cell line panel. We then integrate data from these functional assays and DNA sequencing to improve the selection of functionally relevant genetic alterations. Finally, we probe the association of such FA/HR aberrations with clinical outcome in a well-defined homogenous HNSCC patient cohort (*n* = 77) treated with radiotherapy and cisplatin to test their prognostic value.

## RESULTS

### Hypersensitivity to the DNA crosslinking agent mitomycin C reveals functional crosslink repair defects in HNSCC

**Figure 1:**
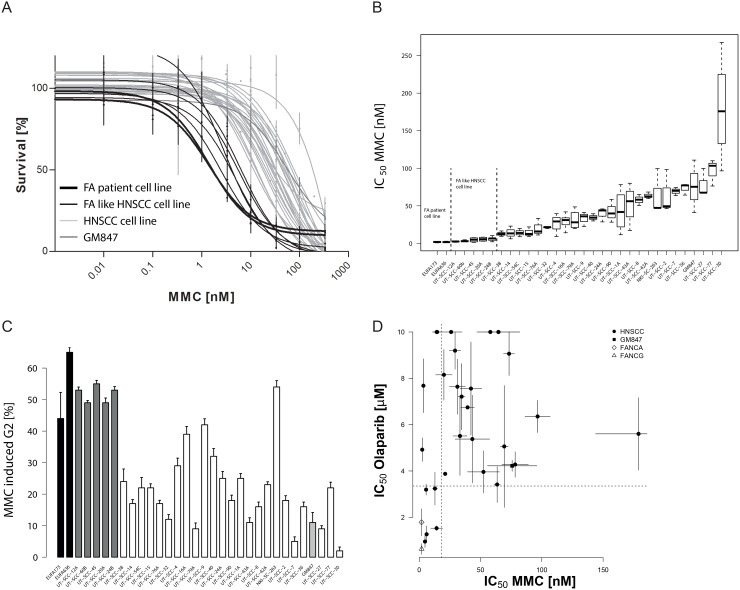
Sensitivity of HNSCC cell lines to mitomycin C and PARP inhibition (**A**) MMC sensitivity as measured by a prolonged growth assay. The average surviving fraction derived from three to five independent experiments per cell line. Errors are SEM. Note, MMC concentrations are log-transformed. A non-linear fit on the log-transformed data is shown. (**B**) Boxplot with MMC IC50 values in the cell line panel. Values are calculated from the curve fits on the individual experiment data and are the average of three to five independent experiments. (**C**) G2/M cell cycle phase arrest 48 hrs after 1 μM MMC treatment. Cell lines are ranked according to their MMC IC50. MMC-induced G2 values are corrected for the untreated. Errors are SEM. (**D**) Comparison of MMC and olaparib sensitivity in the HNSCC cell line panel. The graph demonstrates the lack of MMC-resistant but olaparib hypersensitive cell lines. Olaparib IC50 values were determined on the individual curve-fits of three to five independent experiments. Errors are SEM. “FA-like” have been highlighted for presentation and cross-comparison purposes and depicts HNSCC cell lines with MMC IC50 values that are not significantly different from those of the FA-patient cell lines (EUFA173 and EUFA 636) that served as positive controls.

Hypersensitivity to the crosslinking agent mitomycin C (MMC) and a strong G2 cell cycle block in response to MMC are hallmarks of FA-pathway disruption [24, 25]. To test whether sporadic HNSCCs have such DNA repair defects, we treated 29 HNSCC cell lines with MMC and assessed their survival in long term growth assays. The HNSCC cell lines showed a broad spectrum of sensitivities to MMC (Figure 1A) with IC_50_ values ranging over 50-fold from 5–250 nM (Figure 1B, Supplementary Table 1). MMC-hypersensitivity, in particular if as pronounced as in the FA-patient derived cells, strongly suggests a functional crosslink repair defect in a significant proportion of the cell lines.

To confirm this FA-like phenotype additional functional endpoints were analyzed. We first examined cell cycle progression after treatment with MMC (Figure 1C, Supplementary Figure 1, Supplementary Table 1). Exponentially growing cells were treated with MMC and analyzed by flow-cytometry for cell cycle phase distribution 48 hrs after treatment. Both FA-positive controls showed a strong G2-block. Consistent with the prior analysis, the MMC-hypersensitive UT-SCC-12A, UT-SCC-20A, UT-SCC-24B, UT-SCC-45 and UT-SCC-60B cell lines showed a G2-block that was comparable to the FA-positive controls. The IC_50_ for MMC-induced cell killing correlated strongly with induction of a G2-block (*p* < 0.0005) (Supplementary Figure 1B). Proliferation ultimately exposes the cytotoxicity of MMC-induced DNA crosslinks through replication attempts. This could therefore affect drug sensitivity values in a manner that is unrelated to repair efficiency. However, we found no such association between the MMC-induced cytotoxicity or the G2-block and S-phase content (Supplementary Figure 2), further illustrating the value of individual cell doubling time adaptation and choice of long term survival assay. Taken together, both tests show functional defects in cellular DNA crosslink repair in a significant proportion of HNSCC cell lines.

### Hypersensitivity to the PARP inhibitor olaparib supports functional DNA repair defects

PARP inhibitors have been shown to reveal FA/HR-defects, by inducing kill in repair-defective cells [5, 26]. We therefore tested our HNSCC panel for sensitivity to the PARP inhibitor olaparib (AstraZeneca). Olaparib response varied highly (Supplementary Figure 3). IC_50_ was not reached at the highest tested dose in eight of the cell lines. As reported previously, FA-cells were hypersensitive to olaparib [26] and define the lower limit in this sensitivity range. Several HNSCC cell lines were indistinguishable from these FA-patient derived fibroblasts, strongly indicating functional FA/HR-pathway defects in these lines. No correlation was found between S-phase content or doubling time and olaparib (Supplementary Figure 4A).

We did not find a strong correlation between MMC and olaparib response (Figure 1D, Supplementary Figure 4B). Consistent with the existence of olaparib resistance mechanisms unrelated to or bypassing HR-mediated repair, some MMC-hypersensitive cell lines, the UT-SCC-12A and UT-SCC-60B, did not exhibit a concomitant olaparib hypersensitivity [27]. Notably, all highly olaparib-sensitive cells were also MMC-hypersensitive, further confirming a functional FA/HR-pathway defects in these cell lines.

### Aberrant FANCD2 expression and mono-ubiquitylation point to FA-pathway defects

**Figure 2:**
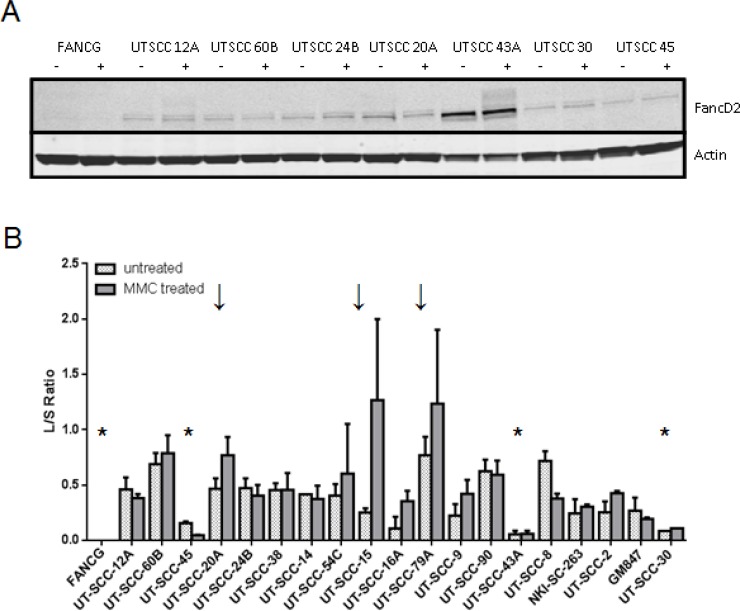
FANCD2-mono-ubiquitylation capacity in the HNSCC panel FANCD2-mono-ubiquitylation ability was assessed by exposure to MMC. (**A**) Representative example of FANCD2-ubiquitylation western blot analyses are shown. Lysates were prepared from untreated (−) or MMC-treated cells (+) 6 h after treatment. The lack of the upper band indicates a lack of the mono-ubiquitylated form of FANCD2 (FANC2-L) and a defect upstream in the Fanconi pathway. Actin served as a loading control. (**B**) Quantification of MMC-induced FANCD2-mono-ubiquitylation in the HNSCC panel. Quantified FANCD2-L/S values in untreated (dotted bars) and MMC-treated (solid bars) samples of each analyzed HNSCC cell line are shown. HNSCC values are ranked according to their MMC sensitivity. Errors are SEM. Stars (*) indicate examples with an overall lack of FANCD2-ubiquitylation, arrows (↓) in contrast depict HNSCC with a pronounced MMC-induced FANCD2 mono-ubiquitylation as expected by a fully functional pathway.

Mono-ubiquitylation of FANCD2 is an essential step in FA-pathway activation upon DNA damage [27, 28]. A lack of MMC-induced FANCD2-L demonstrates FA-pathway defects upstream of the ubiquitylation event. We tested the FANCD2-ubiquitylation capacity in 18 HNSCC cell lines and found that anomalies are common. FANCD2 expression varied and was very high in UT-SCC-43A (Figure 2, Supplementary Figure 5). Only three cell lines responded to MMC treatment with an increase in FANCD2-mono-ubiquitylation. Despite a strong MMC cell survival assay response, UT-SCC-45 and UT-SCC-43A lack efficient FANCD2 mono-ubiquitylation at any condition. These data further support DNA damage response irregularities and FA-complex defects leading to disrupted FA-pathway activation in HNSCC.

### FA expression analysis reveals lack of FANCF expression in one HNSCC cell line

**Figure 3:**
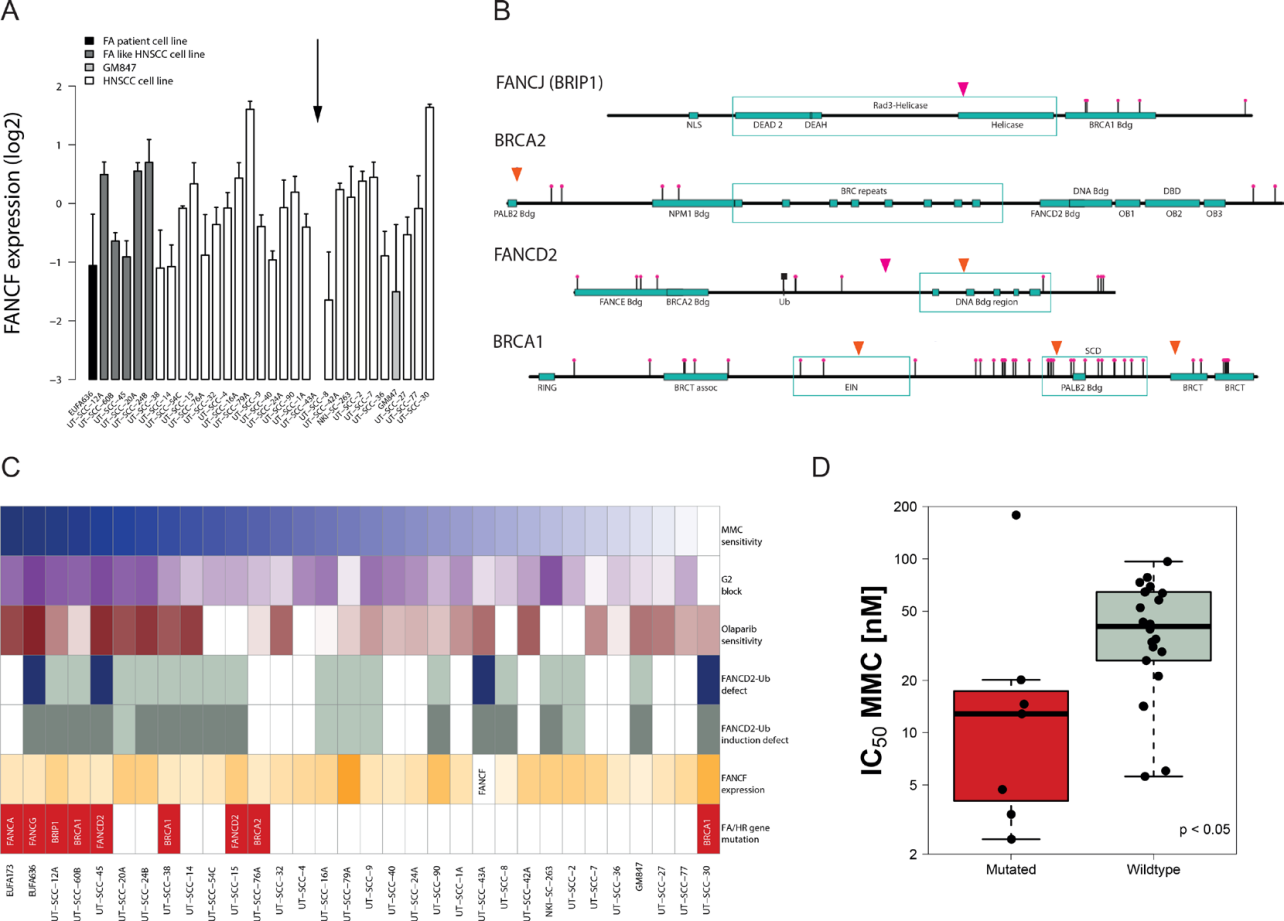
Identification of genetic FA and HR pathway alterations and their association with a functional repair defect (**A**) FANCF expression in the HNSCC cell line panel. HNSCC cell lines are ordered according to their MMC sensitivity. The relative FANCF expression in the individual HNSCC cell lines is shown as a deviation from average (log2-transformed) after normalization to the two housekeeping genes. Arrow (↓) depicts lack of FANCF expression. Errors are SD on the means of 3 to 5 independent PCR reactions. (**B**) Identification of potential FA and HR gene mutations in HNSCC. Homozygous rare sequence variants were found in BRCA1, two in FANCD2 and one in BRIP1 (FANCJ) and BRCA2 in 7 of the 29 HNSCC. Rare SNPs are depicted in orange, unreported non-synonymous variants in pink. (**C**) Comprehensive summary of the HNSCC DNA repair defect data. HNSCC cell lines are ordered according to their ranking in MMC sensitivity (top panel). MMC sensitivity, MMC-induced G2 block and Olaparib sensitivity are represented by a color grading with darker colors representing defects in those parameters. Blue bars display defects in FANCD2 mono-ubiquitylation and grey bars represent a lack of induction by MMC (white bars = not determined). FANCF bars are color-ranked according to their expression values. Red bars demonstrate the identification of DNA sequence variants (as shown in B). (**D**) MMC sensitivity of HNSCC with FA/HR gene variants (in red) compared to HNSCC in which such variants could not be found (*p* < 0.05).

The observed FA phenotype-like properties of a proportion of the HNSCC prompted us to search for the genetic cause. FANCF down-regulation mediated by promoter methylation has been reported in HNSCC [18]. We therefore determined FANCF expression by PCR and tested whether it was associated with the observed functional defects. No correlation or cut-off analysis supported a role of FANCF expression in defining MMC sensitivity (Figure 3A). However, the FANCF expression level was undetectable in UT-SCC-43A and is consistent with its lack of MMC-induced FANCD2-ubiquitylation, thereby revealing the likely cause of the observed FA-pathway defect in this cell line. RNA-sequencing analysis in all cell lines confirmed the lack of FANCF expression in UT-SCC-43A but did not reveal additional hits.

### DNA sequencing identifies FA/HR gene variants with a functional association

To further uncover genetic defects that may explain the above observed crosslink repair defects, we performed capture-based sequencing including 27 canonical FA/HR-pathway genes (Supplementary Table 2). Copy number analysis on the sequencing data did not reveal homozygous deletions of the FA/HR genes. We next called single nucleotide variants and small indels and applied a variant selection protocol that was designed to enrich for functional alterations (Supplementary Table 3). In brief, considering the requirement for loss of heterozygosity (LOH) for most mutated DNA repair genes to affect cellular function, it selects for homozygous non-synonymous variants with a low or lacking minor allele frequency with the aim to enrich for variants and mutations with a potential functional association. Confirming the specificity of this approach, the known *FANCA* and *FANCG* germline mutations in the FA-patient derived fibroblasts were identified and no additional variants were selected. No FA/HR gene variants, as defined by our selection criteria, were found in the GM847 normal fibroblasts.

The analysis revealed seven FA/HR-variants in seven HNSCC lines: three in *BRCA1*, two in *FANCD2* and one in *BRCA2* and *BRIP1* (Figure 3B). With the exception of UT-SCC-30, these variants exclusively occurred in the MMC-hypersensitive cell lines, supporting their potential role in identifying crosslinker sensitivity (Figure 3C). Consistent with an FA/HR defect and the MMC and olaparib hypersensitivity endpoints, the suspected BRCA1 mutation carrier UT-SCC-60B showed impaired radiation-induced rad51 foci formation (Supplementary Figure 6). In contrast, the resistant UT-SCC-30 did not show any apparent HR pathway defects (Supplementary Figure 6), suggesting a false positive assignment by the variant selection protocol. We next used the *in silico* algorithms PolyPhen [29] and SIFT [30] to predict deleteriousness of the variants. Five of these seven variants were predicted to be damaging or deleterious (Supplementary Table 4). While among those variants some are rare or moderately rare SNPs, two of these variants, those in *BRIP1* and *FANCD2*, are unreported in the 1000Genomes database. The location and nature of these variants strongly suggests an impact on protein function. The *BRIP1* variant Gly690Arg is located within the helicase domain and is strongly predicted to affect *BRIP1* protein function (Figure 3B, Supplementary Table 4). The *FANCD2* mutation affects a proline flanking a highly conserved region that encompasses the heterodimer interface, resulting in a deleterious prediction by PolyPhen. The other *FANCD2* missense variant is reported in the 1000 Genomes database and is located within highly conserved regions of the DNA binding domain. It was detected in the FANCD2-mono-ubiquitylation defected UT-SCC-45, indicating a causative link.

LOH is common in tumors of carriers of pathogenic BRCA1/2 germline variants that predispose to breast cancer. This prompted us to investigate LOH in the genes in which the seven variants are located. We therefore assessed the zygosity of all sequenced SNPs in these genes (Supplementary Figure 7). We found that all 19 *BRCA1* SNPs detected in UT-SCC-38 are homozygous, strongly suggesting LOH of the potentially mutated *BRCA1*. Similar evidence of full or partial LOH of *BRIP1* and *BRCA2* is present in the UT-SCC-12A and UT-SCC-76A respectively. Two out of three *BRCA1* variants in UT-SCC-60B are homozygous and have a minor allele frequency (MAF) below 5%, also pointing to potential LOH events. We were not able to assess LOH events using SNPs in the other three cell lines due to low SNP density, however, copy number data point to LOH in the respective genes in two of them, UT-SCC-15 and UT-SCC-45 Supplementary Figure 7).

Some of the selected FA/HR-variants, due to the selection criteria, may just expose LOH events, rather than being causative. Their incidence in the repair-defected cell lines, however, suggests a functional link and therefore a potential role as repair defect markers. Therefore, we evaluated whether all the variants (including possible false positive, i.e. the UT-SCC-30) were associated with MMC-sensitivity. The ‘FA/HR-gene affected’ HNSCC cell lines (i.e. variant-positive) were significantly more sensitive than the non-mutated (Figure 3D, *p* < 0.05). Likewise, MMC-hypersensitive were significantly enriched for FA/HR-pathway variants (Supplementary Figure 8A, *p* < 0.005). This association did not originate from a higher mutation load in these cell lines and was specific to the FA/HR-pathway further supporting the bioinformatics selection approach (manuscript in preparation). We analyzed 17 additional crosslink repair and DNA damage response genes that act in the periphery of the FA/HR-pathways and found one additional rare variant in *ATR* (Supplementary Figure 8B, 8C) in the MMC-sensitive UT-SCC-14, hence improving the MMC-sensitivity association (*p* < 0.01; Supplementary Figure 8D).

Taken together, we identified genetic variants in the FA/HR-pathway that are associated with functional repair defects.

### FA/HR gene variants are present in a considerable proportion of HNSCC tumors

**Figure 4:**
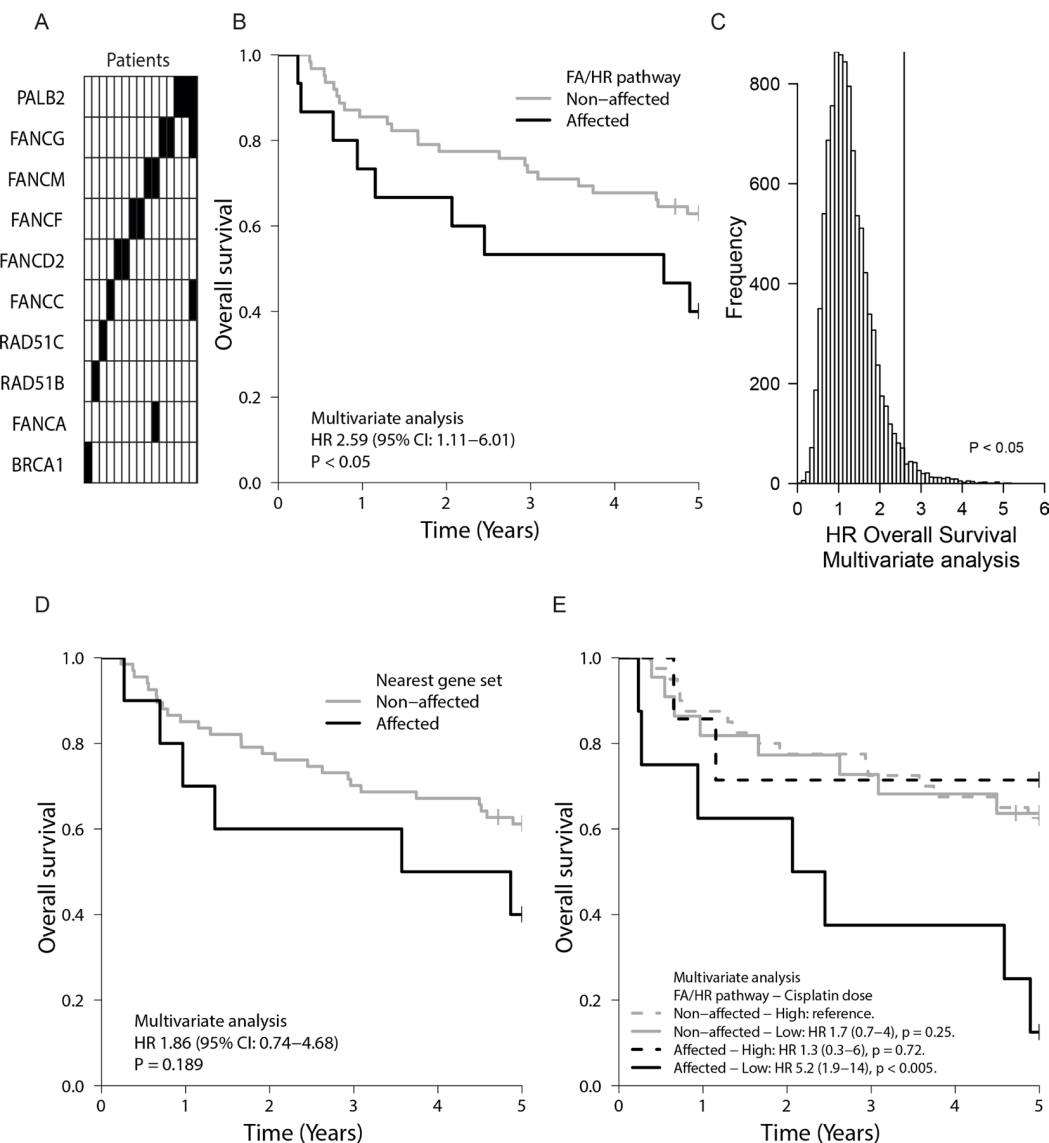
FA/HR gene variants in tumors of HNSCC patients and their prognostic value (**A**) Nineteen FA/HR gene variants were found in fifteen patient samples (columns), resulting in a 19.5% incidence rate (Supplementary Table 5). (**B**) Oro- and hypopharyngeal tumor samples of seventy-seven chemoradiated HNSCC patients were analysed for variants in canonical FA/HR genes by applying the variant selection criteria that returned a functional DNA repair defect association *in vitro*. HNSCC patients with FA/HR gene variants in the tumors (= FA/HR-affected) had a worse overall survival (OS) (*p* < 0.05 in multivariate analysis), demonstrating the impact of potential functional FA/HR repair defects in this patient population. (**C**) Distribution of OS hazard ratios, obtained by repeating the analysis of Figure 4A on ten-thousand randomly selected genes sets of similar base coverage, highlights the significance and specificity of the FA/HR gene variants HR finding. Gene sets were sampled from 529 sequenced cancer-related genes. (**D**) Kaplan–Meier graph showing OS of patients with variants in genes in FA/HR gene chromosomal locations. No significant association was found. (**E**) OS is worst in patients with functionally associating FA/HR gene variants and low cumulative cisplatin dose (HR 5.2, *p* < 0.005). In-figure: multivariate hazard ratios (HR) with confidence intervals (95% CI) from multivariate Cox proportional hazard models that include the tested gene set, tumor site, HPV-status and tumor volume.

Encouraged by the strong association of the FA/HR-variants with the functional crosslink repair defect *in vitro*, we investigated the presence of such variants in a cohort of 77 advanced stage oro- and hypopharyngeal HNSCC tumors from chemo-radiated patients (Table 1). Since associated with function, we applied the variant selection protocol that was used for the cell lines, while correcting for stromal contribution in order to enrich for homozygous variants. 19% (15/77) of the HNSCC, a fraction similar to the cell line panel (24%), possessed such FA/HR-variants (herein termed ‘FA/HR-affected’ tumors). Figure 4A and Supplementary Table 5 summarize and list these variants, their predicted alterations and distribution over the patient cohort. Half of these variants were predicted to be deleterious or damaging by PolyPhen and SIFT or were referenced in COSMIC. We further found a higher prevalence of rs17885240 in our patient cohort than in the general population (4/77 versus MAF = 0.0129; *p* < 0.05). Others have shown a high prevalence of rs17885240 in childhood AML (Supplementary Table 5).

**Table 1:** Demographics of HNSCC patient cohort

Patient characteristics		*N* (%)
Gender	M	55 (71)
	F	22 (29)
Primary site	Oropharynx	49 (64)
	Hypopharynx	28 (36)
T-stage	T1	1 (1)
	T2	13 (17)
	T3	35 (46)
	T4	28 (36)
N-stage	N0	10 (13)
	N1	7 (9)
	N2	52 (68)
	N3	8 (10)
T-volumes	0–30 cc	38 (49)
	>30	39 (51)
Events	Death	32 (42)
	Locoregional Recurrence	10 (13)
HPV	positive	21 (27)
	negative	56 (73)
Smoker	current	46 (60)
	former	19 (25)
	never	4 (5)
	unknown	8 (10)
Alcohol consumption	yes	47 (61)
	former-alcoholic	13 (17)
	never	8 (10)
	unknown	9 (12)
Cisplatin regimen	daily (6 mg/m^2^, 5 weeks)	17 (23)
	3-weekly (100mg/m^2^, 3×)	46 (59)
	weekly (150 mg/m^2^, 4×)	14 (18)
Cumulative cisplatin dose	low (< 300 mg/m^2^)	30 (39)
	high (≥ 300 mg/m^2^)	47 (61)
		**time**
Median age	at diagnosis	58 years (SD = 9, 6)
Median survival	Overall survival	63 months (SD = 39)
	Locoregional control	63 months (SD = 41)

Pretreatment biopsy material from HNSCC tumors of 77 patients was sequenced and tested for functional repair associated FA/HR-variants, as determined in the cell line panel. All patients received concurrent cisplatin-based chemoradiotherapy, with some patients reaching a high cumulative cisplatin dose of ≥300 mg/m^2^.

### FA/HR gene variants with *in vitro* functional association are associated with poor outcome in HNSCC

Due to the functional repair defect association of the FA/HR gene variants *in vitro*, we next questioned whether the identified patients had a different prognosis. The known and reported HNSCC prognostic variables tumor site, tumor volume and HPV-status affect outcome in our patient cohort (Supplementary Figure 9A–9F) [12]. We next compared the clinical outcome of patients with ‘FA/HR-affected’ tumors to the others, while adjusting for these known prognostic variables in a Cox proportional hazards model. We find that the overall survival (OS) of patients with ‘FA/HR-affected’ tumors is lower (multivariate HR 2.6; 95% CI 1.1-6.0; *p* < 0.05) (Figure 4B, Supplementary Table 6). Locoregional control was worse in these patients (univariate HR 2.1; 95% CI 0.5–8.1; *p* = 0.21 and multivariate HR 3.5; 95% CI 0.8–15.2; *p* = 0.09) (Supplementary Figure 10), but this did not reach significance. This was likely due to the small number of locoregional events (*n* = 10) and also prevents meaningful conclusions from multivariate Cox model analyses that aim to account for the three major clinical variables. There was no apparent association between HPV-status and the presence of FA/HR-variants (Supplementary Table 7). Standard somatic FA/HR-variant selection did not reveal any outcome association, further highlighting the value of FA/HR-variant selection criteria and the functional *in vitro* analysis (Supplementary Figure 11).

Next, we evaluated indirect or FA/HR unrelated elements that may have driven the observed association of the FA/HR-affected tumors with poor patient outcome. Mutation load was not of influence, since these tumors have similar variant and mutation frequencies to the other tumors (Supplementary Figure 12). 529 additional HNSCC and cancer related genes were sequenced in these tumors. To confirm specificity to the FA/HR pathways, we then assessed how likely the HR of 2.6 that was found in the ‘FA/HR-affected’, is, by applying the FA/HR gene variant selection protocol to 10.000 random gene sets of similar total base coverage (Figure 4C). Only 304/10.000 random gene sets have a HR > 2.6, confirming the significance of the association between HR/FA-variants and the poor outcome. Finally, our variant selection approach may have marked LOH of larger chromosomal segments associated with poor outcome. We therefore repeated our analysis on other genes in the same chromosomal location and did not find a significant association with overall survival or locoregional control (Figure 4D, Supplementary Figure 10B, Supplementary Tables 6 and 8) further confirming specificity to the FA/HR-pathway genes.

Not all patients received equally high cisplatin doses, either due to discontinuation or a planned low dose cisplatin chemo-radiation scheme. True (functional) FA/HR-pathway disruption would imply an increased tumor sensitivity to crosslinking agents. We therefore further grouped patients according to their received cumulative cisplatin dose (Figure 4E). In line with the prediction, only FA/HR variants harboring tumors responded to a change in cumulative dose and our data show a benefit from high cumulative cisplatin doses for these patients while failing to show this in the non-FA/HR affected. In summary, we find that FA/HR-pathway variants and mutations mark FA/HR repair defects *in vitro*. Identifying such variants in HNSCC patients allowed us to reveal a worse prognosis group, suggesting that such tumor repair defects have an impact on patient outcome.

## DISCUSSION

Prompted by the relevance of genetic HR/FA repair defects in breast cancer and the high incidence of HNSCC in FA-patients [7, 8], we investigated the role of such defects in sporadic HNSCC. As similar genetic markers were missing, we first determined the incidence and properties of functional DNA repair defects by performing multiple functional assays on 29 HNSCC cell lines. We find that a significant proportion exhibit DNA crosslink repair defects comparable to FA-patient derived fibroblasts, as determined by the functional endpoints MMC-hypersensitivity, G2-blocks, olaparib-hypersensitivity and FANCD2 mono-ubiquitylation. Our comprehensive large cell line panel data therefore shows the high prevalence of functional DNA crosslink repair defects in HNSCC *in vitro*. We next determined possible genetic causes with the intention to translate these findings to the clinic and find multiple FA/HR gene variants that are associated with the functional outcome parameters. When retrospectively determining FA/HR gene variants with such an *in vitro* confirmed functional relevance in clinical samples, we find an incidence of 19%. Importantly, patients with tumors that harbor such genetic markers, do worse. No other genetic pathway analysis was able to depict this worse prognosis patient population, thereby further supporting the important role of such potential repair defects in these cases.

Other *in vitro* studies indicated crosslink repair defects in HNSCC. A large variation in response to the crosslinker cisplatin in ten cell lines tested by Snyder *et al.* [31] supports our conclusion. We find at least two HNSCC cell lines that are incapable of mono-ubiquitylating FANCD2 (UT-SCC-45 and UT-SCC-43A) indicating a defect upstream or in FANCD2 itself. In both cell lines this was consistent with the genetic defect. This low incidence is consistent with other smaller studies and explains the lack of disrupted FANCD2 mono-ubiquitylation or FA-gene expression deregulation in the study of Snyder *et al*. Also Burkitt [32] observed defective FANCD2-foci formation in three cisplatin sensitive HNSCC. The authors found decreased BRCA1 expression and showed this caused cisplatin sensitivity in one cell line. Furthermore, MMC and cisplatin-responsive HNSCC cell lines have been found by a chromosomal breakage assay [22]. No other study has comprehensively tested for both functional and genetic FA/HR-defects in such a large panel of HNSCC cell lines. Some studies sought after genetic defects in functional affected only, others employed endpoints, such as FANCD2-ubiquitylation, that do however not capture repair defects downstream of the event.

The discovery of frequent FA/HR defects may have also been impeded by weak IC50 values that were determined by short term metabolic assays in other studies . Our HNSCC cell line panel exhibited a large range of doubling times (17 h to 79 h, Supplementary Table 1) providing sensitivity and specificity issues in standard short term (72 h) survival assays. Our long term and doubling time adjusted survival assay was able to uncover MMC sensitivity in slow HNSCC with DTs of over 48h. Yet, other HR/FA unrelated factors can affect some of the endpoints on an individual basis and therefore demands multiple endpoint analysis. The response to MMC partly depends on metabolic activation by cellular reductases such as the NAD(P)H:Quinone oxireductase-1 (NQO1) and the extent of glutathione detoxification [33, 34]. However, these processes rarely affect the response by an enhancement factor of more than three [35–37]. It should be noted that such factors could also affect the apparent FANCD2-ubiquitylation response at a given MMC concentration. Rad51 foci induction by radiation, however, reflects the ability to engage HR [38]. Consistent with its resistance to MMC and olaparib, this response therefore excludes a BRCA defect in UTSCC-30 (Supplementary Figure 6). Apparent Olaparib resistance is possible in the presence of FA/HR defects. This can be the result of an increased expression of the drug efflux transporters *Abcb1a/b* genes, which encode the P-glycoprotein [27]. Moreover, recent studies show a role for p53-binding protein 1 (53BP1) and REV7 for olaparib resistance in BRCA1 deficient cells [39, 40]. Notably and consistent with our observation, the authors report olaparib resistant tumors which maintained crosslinker cisplatin sensitivity [40]. While FA/HR-defects can therefore not be excluded in olaparib resistant HNSCC or in HNSCC with somewhat higher MMC IC50 values, the manifestation of the combined olaparib and MMC hypersensitivity is a strong indicator of functional crosslink repair defects. By our multiple endpoint analysis we were able to unmask multiple FA/HR defects. At least a quarter of HNSCC exhibited strong FA/HR defects as determined by multiple endpoints. Any cut-off for classification would be however arbitrary, clearly evident from the distribution shown in Figure 1B or Figure 3C and many more HNSCC show signs of repair defects.

Aided by the quantity of different cell lines and endpoint analyses in our study, we were also able to define genetic and repair defect associations ultimately providing a variant selection protocol that was designed to enrich for variants with a potential functional association. The selection of homozygous variants and rare SNPs are noteworthy elements of our variant selection protocol. First, we reasoned that functional DNA repair defects will generally be enabled or caused by the loss of the functional allele and therefore only selected homozygous variants. LOH of three FA genes has recently been reported in sporadic HNSCC (33). As shown here, our approach can also capture and enrich for potentially relevant LOH events in the case of rare SNPs. Thus we acknowledge that these particular variants may not have a direct impact on the gene function. A related challenge in evaluating the consequence of individual missense variants was the absence of matched normal samples. Selection of homozygous variants does also not exclude an effect on repair of potential heterozygous compound mutations which are difficult to evaluate. Second, and different from other studies that discard SNPs, we included SNPs with a MAF < 2.5%. This allows for increased LOH event detection and is also based on our hypothesis that a fraction of HNSCC patients may bear hypomorphic FA/HR germline variants [41] that are pathogenic only when exposed to high levels of crosslinkers. HNSCC patients are often heavy drinkers and smokers and exposed to DNA crosslinking chemicals in both. From demographic data we estimated that 5–10% of all heavy alcohol and tobacco users will develop HNSCC [42, 43]. In breast cancer, a single pathogenic BRCA1/2 variant can reach a penetrance of more than 50% [44]. Hence, we estimate that if HNSCC would be promoted by few SNPs, these SNPs could be present in up to 2.5–5% of the general population, thus used this value as cut-off. These estimates highlight how SNPs with such a relative high MAF could contribute to a HNSCC predisposition but remain masked as they would not harm the majority of carriers with little exposure to the carcinogens. Due to the heavy exposure to crosslinking agents we also expect that the type and nature of “pathogenic” FA/HR variants is largely different from those causing breast cancer or Fanconi Anemia. Indeed, we find FA/HR gene variants that have not been classified as pathogenic in hereditary breast cancer or Fanconi Anemia. Yet, some show indications of a potential disruptive nature (FANCD2 P834A,RAD51C G264S) . Other variants may have simply marked LOH events in these cells and patients. Importantly, the identified variants by the chosen selection criteria were strongly associated with MMC-sensitivity, thus providing a functional link.

Homozygous variant detection is challenging in patient tumor samples due to unknown tumor sample purity, ploidy and intra-tumor heterogeneity [45]. Corrections for stromal contributions were made, using the pathologist's tumor purity estimate. Yet, in tumor samples with high stroma contribution of 40–50%, concessions had to be made ultimately allowing for a higher degree of false positives with regards to the homozygous status of the selected variants. However, when analyzing genetic data of 77 HNSCC tumors, and classifying patients according to tumor FA/HR-variant presence as FA/HR-affected we found that patients with such tumors had a worse prognosis. This worse outcome is analogous to reports in breast cancer studies, in which confirmed pathogenic BRCA1/2 mutations were associated with a more malignant phenotype and a worse prognosis [46, 47]. Chromosomal and genetic instability promoted by the DNA repair defect and the associated tumor heterogeneity could be the driving force for the malignant phenotype and the apparent treatment failure in such patients. Since such tumors are hypersensitive to crosslinking agents *in vitro*, future studies will have to determine whether this specific patient group do benefit from crosslinker-based treatment. HNSCC patients benefit from high cumulative cisplatin doses [48]. Our preliminary analysis on our initial cohort data shows that this benefit may very well be based on tumor DNA repair defects (Figure 4E). Although belonging to a poor prognosis group, the patients with FA/HR variants containing tumors were the only ones who appeared to benefit from cisplatin dose intensification (high dose vs low dose) thereby establishing survival rates as high as in the other patients.

The accomplishment of personalized medicine requires the discovery and identification, by functional and genomic approaches, of processes that could define treatment options. We assessed, quantified and characterized the FA/HR DNA repair pathway defects and probed their clinical relevance. FA/HR genomic variant selection that was supported by the functional *in vitro* analysis helped to reveal an association with poor survival in a cohort of chemo-radiated HNSCC patients and thereby points to the prognostic value of DNA repair defect identification in cancer. HNSCC has a dismal prognosis, particularly when diagnosed at an advanced state. Our data stress the relevance of repair defects in establishing such bad prognosis and reveal clinical treatment options at the same time since these defects are associated with the benefit of high cumulative doses of crosslinking agents.

Together, our data suggest a novel role of FA/HR-pathway aberrations in both sporadic HNSCC etiology and prognosis. To our knowledge this is also the first report that shows how a comprehensive *in vitro* functional and genetic DNA sequencing based pathway analysis can reveal or enrich for functional and therefore relevant genetic alterations, thereby educating clinical marker selection strategies for biomarker development. The study enabled us to depict a subpopulation of patients with a bad prognosis that might be associated with genomic instability features through the depicted DNA repair defects. In the context of precision medicine, these defects can be exploited for tumor-targeted therapy options (e.g. PARP inhibitors) or personalized cancer treatment with crosslinking agents [6, 49–51].

## MATERIALS AND METHODS

### Cell lines and cell culture

Head and neck cancer cell lines were established at the University Hospital in Turku (UT-SCC; listed in Supplementary Table 1), Finland and at the Netherlands Cancer Institute (NKI-SCC-263) [52–54]. 23 HNSCC are TP53 mutated and only UT-SCC-45 is confirmed TP53 wildtype. Cells were grown and assayed under low oxygen (4%) conditions and were cultured under standard culture condition at 37° C and 5% CO_2_ in DMEM supplemented with 10% fetal bovine serum, 1% penicillin/streptomycin and 1% non-essential amino acids (Invitrogen).

### Patients and clinical cohort

Pre-treatment tumor samples were obtained by biopsy from patients enrolled in our hospital from 2001 to 2010 and after documented informed consent. Patient and tumor characteristics are listed in Table 1. All patients were treated with concurrent cisplatin-based chemoradiotherapy (70 Gy in 35 fractions). Different cisplatin regimens were administered: daily (6 mg/m^2^ intravenously), three-weekly (100 mg/m^2^ intravenously), or weekly for the first 4 weeks of radiotherapy (150 mg/m^2^ intra-arterial). Final cumulative cisplatin doses were recorded and patients were categorized into low (<300 mg/m^2^) and high (≥300 mg/m^2^) dose categories (Table 1). Tumor volumes were assessed on RT treatment planning CTs. Two categories (0–30 and >30 cc) were established and considered in multivariate analyses. Time to locoregional recurrence was calculated from the start of treatment until the first of the following events: local or regional recurrence (event), death or last follow-up (censored). Overall survival time was calculated until death (event) or until the last follow-up (censored). HPV status was determined by capture-based sequencing and validated using p16 and p53 IHC and PCR on suspected positive cases.

### G2 block analysis

Cells were cultured in 6-well plates for three days and treated with the indicated doses of MMC (Sigma-Aldrich) for 2 hours. After MMC treatment, cells were washed with PBS and incubated with fresh non-drug-containing medium. 48hrs later cells were prepared for flow-cytometry analysis (FAC Scan, Becton Dickinson, San Jose California USA) and re-suspended in PBS containing PI (propidium iodide, 10 μg/ml) and RNAse (0,02 mg/ml). Cell cycle phase distributions were measured on the PI histograms using the software CellQuest (Becton Dickinson).

### MMC sensitivity analysis

Cultures were exposed to different concentrations of MMC (Sigma-Aldrich) after 1 day of culture. Cultures grew until the untreated cells had undergone at least five population doublings. Sub-confluent status was tested with internal linearity controls. Live cells from 3–6 sub-confluent wells were counted with a CASY cell counter (Schärfe system, Scotch plains, New Jersey). Survival is the fraction of the number of treated to untreated cells in %. IC_50_ values (MMC concentration with 50% growth inhibition) were calculated from third-order polynomial curve-fits on the growth inhibition values of the individual experiments.

### PARP inhibitor sensitivity analysis

PARP inhibitor olaparib (formerly AZD2281/KU-0059436; provided by AstraZeneca) sensitivity was determined by a long term growth assay using the CyQuant-Cell-Proliferation Assay Kit (Invitrogen) that measures DNA content after cell lysis. Cells were cultured for one doubling-time before exposure to various doses of olaparib while adjusting DMSO concentration in all. Cells were then cultured for a period of at least 5 population doublings. Internal plate controls test linearity and assured sub-confluent status within each experiment. Survival was determined as the fraction of the value in the treated samples compared to the untreated using a TECAN-infinite fluorescence plate reader. IC_50_ values were determined from polynomial curve-fits on the data.

### FANCD2 mono-ubiquitylation analysis

Cells were treated for six hours with or without 100nM MMC two days after plating and harvested for lysate preparation. Proteins were extracted, resolved by SDS-PAGE using a gradient gel (4–15%; Bio-Rad Laboratories) and detected by anti-FANCD2 (1:500 (FI17) Santa Cruz Biotechnology, Santa Cruz) or anti-β-actin IgG (Sigma, 1:50.000), IRDYE 680/800 conjugated anti-mouse or anti-rabbit IgG (Licor, 1:7500). Quantitative analysis of FANCD2-L (long and mono-ubiquitylated) and FANCD2-S (short) of two to three independent experiments on multiple blots each was done on the LICOR platform (Biosciences) and expressed as L/S ratio.

### FANCF expression analysis

RNA from exponentially growing cells was isolated according to standard protocols. FANCF RT-PCR analysis was performed in the 7500 Fast Real-Time PCR system (Applied Biosystems) using the primers listed in Supplementary Table 9. Samples were assayed in triplicate in at least 3 independent RT-PCR reactions and runs. FANCF expression values were normalized to two housekeeping genes. The 7500 Fast system SDS software and the 2-(ddCt) method was used for data analysis. FANCF expression in UT-SCC-43 was undetectable in all independent samples despite high RNA quality values (RIN10) and average housekeeping gene expression. The lower limit for detection was assigned to this line for calculation purposes.

### HNSCC material and DNA isolation

Cellular DNA of the HNSCC cell lines and from fresh frozen tumor material was isolated using the Qiagen AllPrep DNA/RNA Mini Kit. Only material with an average tumor content of 50% and higher, as determined by a pathologist on H&E sections adjacent to and in the midst of the sections collected for DNA sequencing, was included. Researchers and bioinformaticians were blinded to patient information and outcome data. Clinicians were blinded to sequencing data. Clinical variables data and anonymous outcome data were applied after sequencing and variant selection.

### DNA capture and sequencing

Paired-end (PE) fragment libraries were prepared using a genomic DNA library preparation kit (Illumina). The libraries were hybridized to a SureSelect custom-based bait library (Agilent) designed to capture exonic regions (with a 50 bp extension on both sides). After washing, the captured DNA was amplified. Enriched libraries were barcoded, pooled and sequenced on a GAII (Illumina Hiseq-2000) using a 2 × 75 bp PE protocol. Sequencing reads were aligned to the GRCh37.55 Ensembl human reference genome using the Burrows-Wheeler Aligner 0.5.10 backtrack algorithm. Potential PCR duplicates were removed using picard-tools MarkDuplicates. An average read depth of 247 in the cell line samples and 255 in the tumor samples was achieved. Copy numbers were inferred from the DNA-seq data using PropSeg on the cell line panel and CNVkit in the tumor cohort [55, 56].

### Variant and mutation calling

Variants, single nucleotide variants (SNVs) and indels, were called with VarScan 2.3.9 using Samtools mpileup 0.1.19 [57]. Next, we annotated these variants with the RefSeq and 1000 Genomes august 2015 databases using Annovar version date 11-05-2016 [58]. CADD [59], PolyPhen [29], REVEL [60] and SIFT [30] were used for *in silico* variant effect predictions. Supplementary Table 3 describes the filtering steps of the variant selection protocol that was designed to enrich for variants with a functional impact. In brief, variant selection considered variant allele frequency (VAF > 0.8) and rare SNPs (MAF < 2.5%). While maintaining VAF criteria for selection, VAF values in the tumor samples were adjusted to correct for the stromal contribution. Taking into account the pathologist's tumor fraction assessment (TF), the VAF values from the sequencing data were corrected as follows to compute a VAF value for the tumor (VAF_T_):

$$VAF_{T}=\frac{VAF-VAF_{S}\times \left(1-TF\right)}{TF}$$

Matched normal tissue blood samples were largely unavailable. Stromal VAF (VAF_S_) was therefore set to 0.2, thereby providing a minimal VAF value for heterozygosity.

### Statistics

All analyses were performed in the R environment for statistical computing. The MMC IC_50_ values of FA/HR-variant positive versus negative cell lines were compared using a Mann-Whitney *U* test. A two-by-two Table holding the FA/HR-variant status of the third most MMC sensitive cell lines (*n* = 10) versus the other cell lines (*n* = 19) was analyzed with Fisher's exact test. Overall survival, locoregional control and Kaplan–Meier estimators were calculated. Multivariate Cox proportional hazard models included the tested gene set of interest and the clinical covariates tumor site, HPV-status and tumor volume. These clinical covariates have prognostic significance in HNSCC and reached significance in univariate analyses in our cohort. Further statistical analyses and values are accordingly specified in the Supplementary Data.

## SUPPLEMENTARY MATERIALS FIGURES AND TABLES







## Abbreviations

BRCA1/2: Breast Cancer Type 1 Susceptibility Protein 1 or 2
CT: computed tomography scans
FA: Fanconi Anemia
H&E: hematoxylin and eosin
HNSCC: Head and Neck squamous cell carcinoma
HPV: Human papillomavirus
HR: hazard ratio
HR: Homologous recombination
IC50: half maximal inhibitory concentration
IHC: Immunohistochemistry
MAF: Minor allele frequency
MMC: Mitomycin C
OS: Overall Survival
PARP: Poly(ADP-Ribose) Polymerase
PE: paired-end
RT: radiotherapy
SDS-PAGE: Sodium Dodecyl Sulfate Polyacrylamide Gel Electrophoresis
SNV: single nucleotide variant
TF: tumor fraction
UT-SCC: University Turku - Squamous Cell Carcinoma
VAF: variant allele frequency

## References

[1] Kim H, D’Andrea AD (2012). Regulation of DNA cross-link repair by the Fanconi anemia/BRCA pathway. Genes Dev.

[2] Mateo J, Carreira S, Sandhu S, Miranda S, Mossop H, Perez-Lopez R, Nava Rodrigues D, Robinson D, Omlin A, Tunariu N, Boysen G, Porta N, Flohr P (2015). DNA-repair defects and olaparib in metastatic prostate cancer. N Engl J Med.

[3] Moschetta M, George A, Kaye SB, Banerjee S (2016). BRCA somatic mutations and epigenetic BRCA modifications in serous ovarian cancer. Ann Oncol.

[4] Helleday T (2010). Homologous recombination in cancer development, treatment and development of drug resistance. Carcinogenesis.

[5] Farmer H, McCabe N, Lord CJ, Tutt AN, Johnson DA, Richardson TB, Santarosa M, Dillon KJ, Hickson I, Knights C, Martin NM, Jackson SP, Smith GC, Ashworth A (2005). Targeting the DNA repair defect in BRCA mutant cells as a therapeutic strategy. Nature.

[6] O’Connor MJ (2015). Targeting the DNA damage response in cancer. Mol Cell.

[7] Rosenberg PS, Alter BP, Ebell W (2008). Cancer risks in Fanconi anemia: findings from the German Fanconi Anemia Registry. Haematologica.

[8] Kutler DI, Auerbach AD, Satagopan J, Giampietro PF, Batish SD, Huvos AG, Goberdhan A, Shah JP, Singh B (2003). High incidence of head and neck squamous cell carcinoma in patients with Fanconi anemia. Arch Otolaryngol Head Neck Surg.

[9] Kamangar F, Dores GM, Anderson WF (2006). Patterns of cancer incidence, mortality, and prevalence across five continents: defining priorities to reduce cancer disparities in different geographic regions of the world. J Clin Oncol.

[10] Leemans CR, Braakhuis BJ, Brakenhoff RH (2011). The molecular biology of head and neck cancer. Nat Rev Cancer.

[11] Pignon JP, le Maître A, Maillard E, Bourhis J, and MACH-NC Collaborative Group (2009). Meta-analysis of chemotherapy in head and neck cancer (MACH-NC): an update on 93 randomised trials and 17,346 patients. Radiother Oncol.

[12] Baumann M, Krause M, Overgaard J, Debus J, Bentzen SM, Daartz J, Richter C, Zips D, Bortfeld T (2016). Radiation oncology in the era of precision medicine. Nat Rev Cancer.

[13] Linge A, Löck S, Gudziol V, Nowak A, Lohaus F, von Neubeck C, Jütz M, Abdollahi A, Debus J, Tinhofer I, Budach V, Sak A, Stuschke M, and DKTK-ROG Low (2016). Cancer Stem Cell Marker Expression and Low Hypoxia Identify Good Prognosis Subgroups in HPV(−) HNSCC after Postoperative Radiochemotherapy: A Multicenter Study of the DKTK-ROG. Clin Cancer Res.

[14] Begg AC, Stewart FA, Vens C (2011). Strategies to improve radiotherapy with targeted drugs. Nat Rev Cancer.

[15] Brooks PJ, Theruvathu JA (2005). DNA adducts from acetaldehyde: implications for alcohol-related carcinogenesis. Alcohol.

[16] Barón AE, Franceschi S, Barra S, Talamini R, La Vecchia C (1993). A comparison of the joint effects of alcohol and smoking on the risk of cancer across sites in the upper aerodigestive tract. Cancer Epidemiol Biomarkers Prev.

[17] Wreesmann VB, Estilo C, Eisele DW, Singh B, Wang SJ (2007). Downregulation of Fanconi anemia genes in sporadic head and neck squamous cell carcinoma. ORL J Otorhinolaryngol Relat Spec.

[18] Marsit CJ, Liu M, Nelson HH, Posner M, Suzuki M, Kelsey KT (2004). Inactivation of the Fanconi anemia/BRCA pathway in lung and oral cancers: implications for treatment and survival. Oncogene.

[19] Sparano A, Quesnelle KM, Kumar MS, Wang Y, Sylvester AJ, Feldman M, Sewell DA, Weinstein GS, Brose MS (2006). Genome-wide profiling of oral squamous cell carcinoma by array-based comparative genomic hybridization. Laryngoscope.

[20] Network CG, and Cancer Genome Atlas Network (2015). Comprehensive genomic characterization of head and neck squamous cell carcinomas. Nature.

[21] Morris LG, Chandramohan R, West L, Zehir A, Chakravarty D, Pfister DG, Wong RJ, Lee NY, Sherman EJ, Baxi SS, Ganly I, Singh B, Shah JP (2016). The molecular landscape of recurrent and metastatic head and neck cancers: insights from a precision oncology sequencing platform. JAMA Oncol.

[22] Stoepker C, Ameziane N, van der Lelij P, Kooi IE, Oostra AB, Rooimans MA, van Mil SE, Brink A, Dietrich R, Balk JA, Ylstra B, Joenje H, Feller SM, Brakenhoff RH (2015). Defects in the Fanconi Anemia pathway and chromatid cohesion in head and neck cancer. Cancer Res.

[23] Romick-Rosendale LE, Lui VW, Grandis JR, Wells SI (2013). The Fanconi anemia pathway: repairing the link between DNA damage and squamous cell carcinoma. Mutat Res.

[24] Akkari YM, Bateman RL, Reifsteck CA, D’Andrea AD, Olson SB, Grompe M (2001). The 4N cell cycle delay in Fanconi anemia reflects growth arrest in late S phase. Mol Genet Metab.

[25] Sasaki MS, Tonomura A (1973). A high susceptibility of Fanconi's anemia to chromosome breakage by DNA cross-linking agents. Cancer Res.

[26] McCabe N, Turner NC, Lord CJ, Kluzek K, Białkowska A, Swift S, Giavara S, O’Connor MJ, Tutt AN, Zdzienicka MZ, Smith GC, Ashworth A (2006). Deficiency in the repair of DNA damage by homologous recombination and sensitivity to poly(ADP-ribose) polymerase inhibition. Cancer Res.

[27] Rottenberg S, Jaspers JE, Kersbergen A, van der Burg E, Nygren AO, Zander SA, Derksen PW, de Bruin M, Zevenhoven J, Lau A, Boulter R, Cranston A, O’Connor MJ (2008). High sensitivity of BRCA1-deficient mammary tumors to the PARP inhibitor AZD2281 alone and in combination with platinum drugs. Proc Natl Acad Sci U S A.

[28] Garcia-Higuera I, Taniguchi T, Ganesan S, Meyn MS, Timmers C, Hejna J, Grompe M, D’Andrea AD (2001). Interaction of the Fanconi anemia proteins and BRCA1 in a common pathway. Mol Cell.

[29] Adzhubei IA, Schmidt S, Peshkin L, Ramensky VE, Gerasimova A, Bork P, Kondrashov AS, Sunyaev SR (2010). A method and server for predicting damaging missense mutations. Nat Methods.

[30] Ng PC, Henikoff S (2003). SIFT: predicting amino acid changes that affect protein function. Nucleic Acids Res.

[31] Snyder ER, Ricker JL, Chen Z, Waes CV (2007). Variation in cisplatinum sensitivity is not associated with Fanconi Anemia/BRCA pathway inactivation in head and neck squamous cell carcinoma cell lines. Cancer Lett.

[32] Burkitt K, Ljungman M (2007). Compromised Fanconi anemia response due to BRCA1 deficiency in cisplatin-sensitive head and neck cancer cell lines. Cancer Lett.

[33] Seow HA, Penketh PG, Baumann RP, Sartorelli AC (2004). Bioactivation and resistance to mitomycin C. Methods Enzymol.

[34] Tomasz M (1995). Mitomycin C: small, fast and deadly (but very selective). Chem Biol.

[35] Volpato M, Phillips RM (2007). Tailoring targeted therapy to individual patients: lessons to be learnt from the development of mitomycin C. Cancer Genomics Proteomics.

[36] Sawamura AO, Aoyama T, Tamakoshi K, Mizuno K, Suganuma N, Kikkawa F, Tomoda Y (1996). Transfection of human cytochrome P-450 reductase cDNA and its effect on the sensitivity to toxins. Oncology.

[37] Belcourt MF, Hodnick WF, Rockwell S, Sartorelli AC (1996). Differential toxicity of mitomycin C and porfiromycin to aerobic and hypoxic Chinese hamster ovary cells overexpressing human NADPH: cytochrome c (P-450) reductase. Proc Natl Acad Sci U S A.

[38] Naipal KA, Verkaik NS, Ameziane N, van Deurzen CH, Ter Brugge P, Meijers M, Sieuwerts AM, Martens JW, O’Connor MJ, Vrieling H, Hoeijmakers JH, Jonkers J, Kanaar R (2014). Functional ex vivo assay to select homologous recombination-deficient breast tumors for PARP inhibitor treatment. Clin Cancer Res.

[39] Xu G, Chapman JR, Brandsma I, Yuan J, Mistrik M, Bouwman P, Bartkova J, Gogola E, Warmerdam D, Barazas M, Jaspers JE, Watanabe K, Pieterse M (2015). REV7 counteracts DNA double-strand break resection and affects PARP inhibition. Nature.

[40] Jaspers JE, Kersbergen A, Boon U, Sol W, van Deemter L, Zander SA, Drost R, Wientjens E, Ji J, Aly A, Doroshow JH, Cranston A, Martin NM (2013). Loss of 53BP1 causes PARP inhibitor resistance in Brca1-mutated mouse mammary tumors. Cancer Discov.

[41] Chandrasekharappa SC, Chinn SB, Donovan FX, Chowdhury NI, Kamat A, Adeyemo AA, Thomas JW, Vemulapalli M, Hussey CS, Reid HH, Mullikin JC, Wei Q, Sturgis EM (2017). Assessing the spectrum of germline variation in Fanconi anemia genes among patients with head and neck carcinoma before age 50. Cancer.

[42] Ferreira Antunes JL, Toporcov TN, Biazevic MG, Boing AF, Scully C, Petti S (2013). Joint and independent effects of alcohol drinking and tobacco smoking on oral cancer: a large case-control study. PLoS One.

[43] Hashibe M, Brennan P, Benhamou S, Castellsague X, Chen C, Curado MP, Dal Maso L, Daudt AW, Fabianova E, Fernandez L, Wünsch-Filho V, Franceschi S, Hayes RB (2007). Alcohol drinking in never users of tobacco, cigarette smoking in never drinkers, and the risk of head and neck cancer: pooled analysis in the International Head and Neck Cancer Epidemiology Consortium. J Natl Cancer Inst.

[44] Group AB, and Anglian Breast Cancer Study Group (2000). Prevalence and penetrance of BRCA1 and BRCA2 mutations in a population-based series of breast cancer cases. Br J Cancer.

[45] Carter SL, Cibulskis K, Helman E, McKenna A, Shen H, Zack T, Laird PW, Onofrio RC, Winckler W, Weir BA, Beroukhim R, Pellman D, Levine DA (2012). Absolute quantification of somatic DNA alterations in human cancer. Nat Biotechnol.

[46] Goodwin PJ, Phillips KA, West DW, Ennis M, Hopper JL, John EM, O’Malley FP, Milne RL, Andrulis IL, Friedlander ML, Southey MC, Apicella C, Giles GG, Longacre TA (2012). Breast cancer prognosis in BRCA1 and BRCA2 mutation carriers: an International Prospective Breast Cancer Family Registry population-based cohort study. J Clin Oncol.

[47] van den Broek AJ, Schmidt MK, van 't Veer LJ, Tollenaar RA, van Leeuwen FE (2015). Worse breast cancer prognosis of BRCA1/BRCA2 mutation carriers: what's the evidence? A systematic review with meta-analysis. PLoS One.

[48] Strojan P, Vermorken JB, Beitler JJ, Saba NF, Haigentz M, Bossi P, Worden FP, Langendijk JA, Eisbruch A, Mendenhall WM, Lee AW, Harrison LB, Bradford CR (2016). Cumulative cisplatin dose in concurrent chemoradiotherapy for head and neck cancer: A systematic review. Head Neck.

[49] Verhagen CV, de Haan R, Hageman F, Oostendorp TP, Carli AL, O’Connor MJ, Jonkers J, Verheij M, van den Brekel MW, Vens C (2015). Extent of radiosensitization by the PARP inhibitor olaparib depends on its dose, the radiation dose and the integrity of the homologous recombination pathway of tumor cells. Radiother Oncol.

[50] Kirova YM, Savignoni A, Sigal-Zafrani B, de La Rochefordiere A, Salmon RJ, This P, Asselain B, Stoppa-Lyonnet D, Fourquet A (2010). Is the breast-conserving treatment with radiotherapy appropriate in BRCA1/2 mutation carriers? Long-term results and review of the literature. Breast Cancer Res Treat.

[51] Vollebergh MA, Lips EH, Nederlof PM, Wessels LF, Wesseling J, Vd Vijver MJ, de Vries EG, van Tinteren H, Jonkers J, Hauptmann M, Rodenhuis S, Linn SC (2014). Genomic patterns resembling BRCA1- and BRCA2-mutated breast cancers predict benefit of intensified carboplatin-based chemotherapy. Breast Cancer Res.

[52] Joenje H, Levitus M, Waisfisz Q, D’Andrea A, Garcia-Higuera I, Pearson T, van Berkel CG, Rooimans MA, Morgan N, Mathew CG, Arwert F (2000). Complementation analysis in Fanconi anemia: assignment of the reference FA-H patient to group A. Am J Hum Genet.

[53] Demuth I, Wlodarski M, Tipping AJ, Morgan NV, de Winter JP, Thiel M, Gräsl S, Schindler D, D’Andrea AD, Altay C, Kayserili H, Zatterale A, Kunze J (2000). Spectrum of mutations in the Fanconi anaemia group G gene, FANCG/XRCC9. Eur J Hum Genet.

[54] Lansford CD, Grenman R, Bier H, Somers KD, Kim SY, Whiteside TL, Clayman GL, Welkoborsky HJ, Carey TE (1999). Head and neck cancers. Human cell culture.

[55] Rigaill GJ, Cadot S, Kluin RJ, Xue Z, Bernards R, Majewski IJ, Wessels LF (2012). A regression model for estimating DNA copy number applied to capture sequencing data. Bioinformatics.

[56] Talevich E, Shain AH, Botton T, Bastian BC (2016). CNVkit: genome-wide copy number detection and visualization from targeted DNA sequencing. PLOS Comput Biol.

[57] Li H, Handsaker B, Wysoker A, Fennell T, Ruan J, Homer N, Marth G, Abecasis G, Durbin R, and 1000 Genome Project Data Processing Subgroup (2009). The Sequence Alignment/Map format and SAMtools. Bioinformatics.

[58] Wang K, Li M, Hakonarson H (2010). ANNOVAR: functional annotation of genetic variants from high-throughput sequencing data. Nucleic Acids Res.

[59] Kircher M, Witten DM, Jain P, O’Roak BJ, Cooper GM, Shendure J (2014). A general framework for estimating the relative pathogenicity of human genetic variants. Nat Genet.

[60] Ioannidis NM, Rothstein JH, Pejaver V, Middha S, McDonnell SK, Baheti S, Musolf A, Li Q, Holzinger E, Karyadi D, Cannon-Albright LA, Teerlink CC, Stanford JL (2016). REVEL: An Ensemble Method for Predicting the Pathogenicity of Rare Missense Variants. Am J Hum Genet.

