# Supplementary material for: Fanconi anemia and homologous recombination gene variants are associated with functional DNA repair defects in vitro and poor outcome in patients with advanced head and neck squamous cell carcinoma
Source: Oncotarget. 2018 Apr 6;9(26):18198–213. doi: 10.18632/oncotarget.24797 (PMC5915066; doi:10.18632/oncotarget.24797)
Supplement: Supplementary file 1 [file oncotarget-09-18198-s001.pdf]

Fanconi anemia and homologous recombination gene variants are associated with functional DNA repair defects *in vitro* and poor outcome in patients with advanced head and neck squamous cell carcinoma

SUPPLEMENTARY MATERIALS

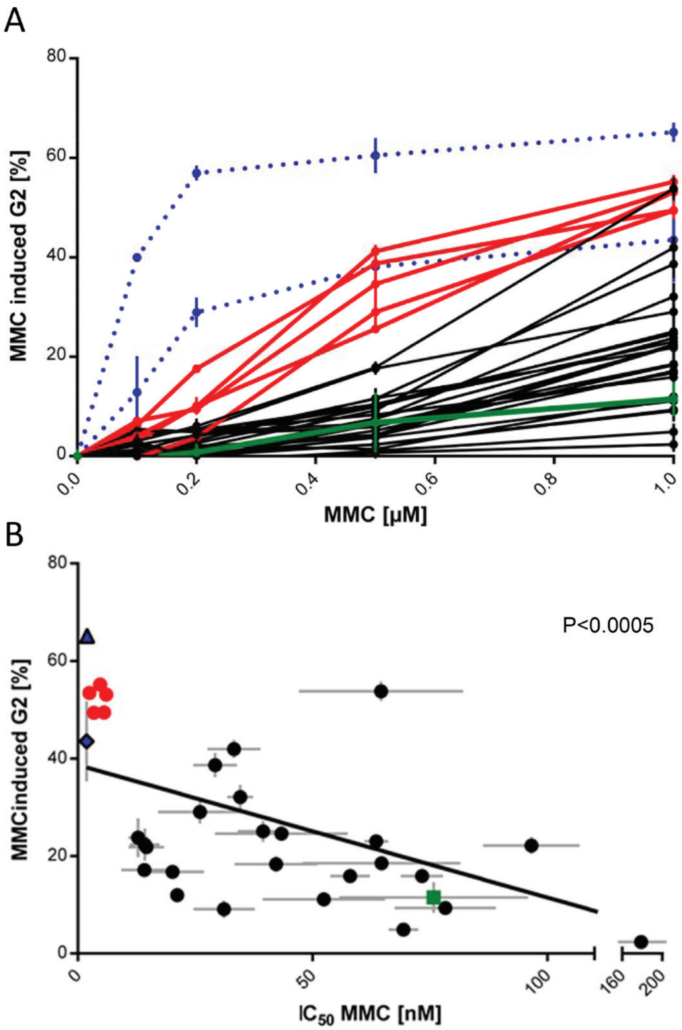

Supplementary Figure 1: MMC-induced G2/M cell cycle phase arrest in HNSCC cell lines and G2 block association with MMC survival. (**A**) G2 phase cell values in % 48hrs after MMC treatment at indicated MMC concentrations. MMC-induced G2 block values are corrected for the untreated cells. Errors are SEM. (**B**) Correlation of MMC sensitivity and MMC-induced G2 block within the HNSCC panel. The IC50 values of the MMC sensitivity correlated significantly with the MMC-induced G2 block (percentage of cells in G2 after 1 µM MMC) with $p < 0.0005$. Initial experiments at 24, 48 and 72 h defined the optimal time point for MMC-induced G2 block analysis in these cell lines. Considering the doubling times, 48 h was chosen for comparison throughout the cell line panel. Lack of G2 blocks in cell lines with considerable long doubling times has been confirmed by analyzing later time points. Untreated, the cell lines varied in the G2 cell cycle phase fraction from 10 to 20%. Two Fanconi Anemia patient-derived fibroblast cell lines with mutations in FANCA and FANCG (EUFA173 and EUFA 636, in blue) serve as positive controls and are compared to a human fibroblast cell line (GM847, green). HNSCC cell lines defined as most FA-like as described in the manuscript are depicted in red.

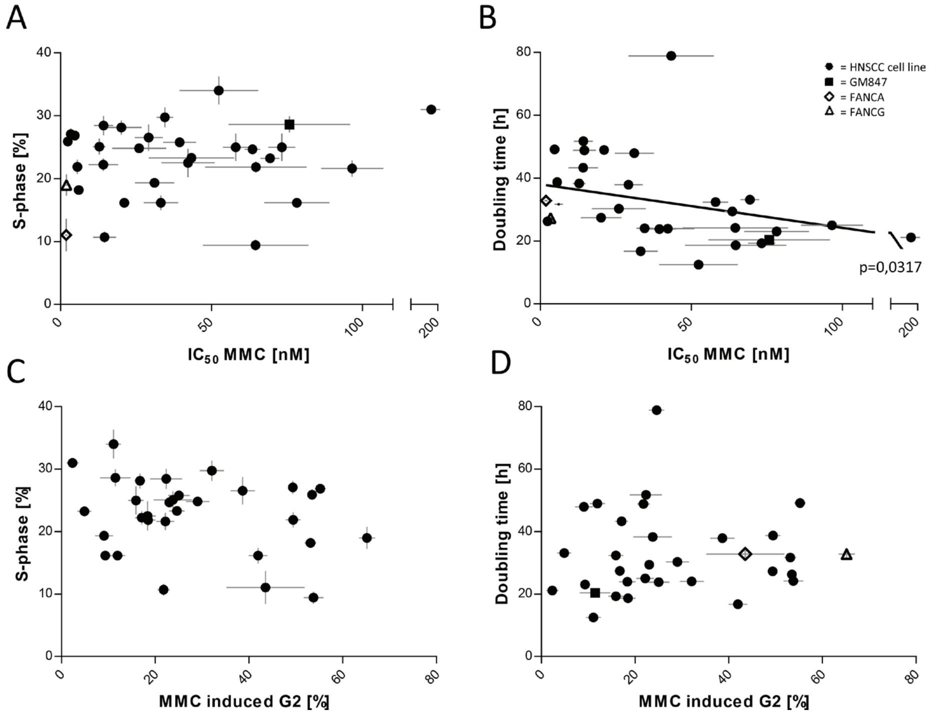

Supplementary Figure 2: Association of MMC response with cell line characteristics. Comparison of MMC sensitivity and MMC-induced G2 block to S-phase content in the cell lines (**A** and **C**, respectively) or to the doubling times (**B** and **D**, respectively). MMC-induced G2 at 1 µM, MMC IC50 and olaparib IC50 values were compared to S-phase content and doubling times in the HNSCC (filled symbols) and assessed for correlation. The controls (open symbols) have been excluded for regression and significance analyses. Error bars are SEM.

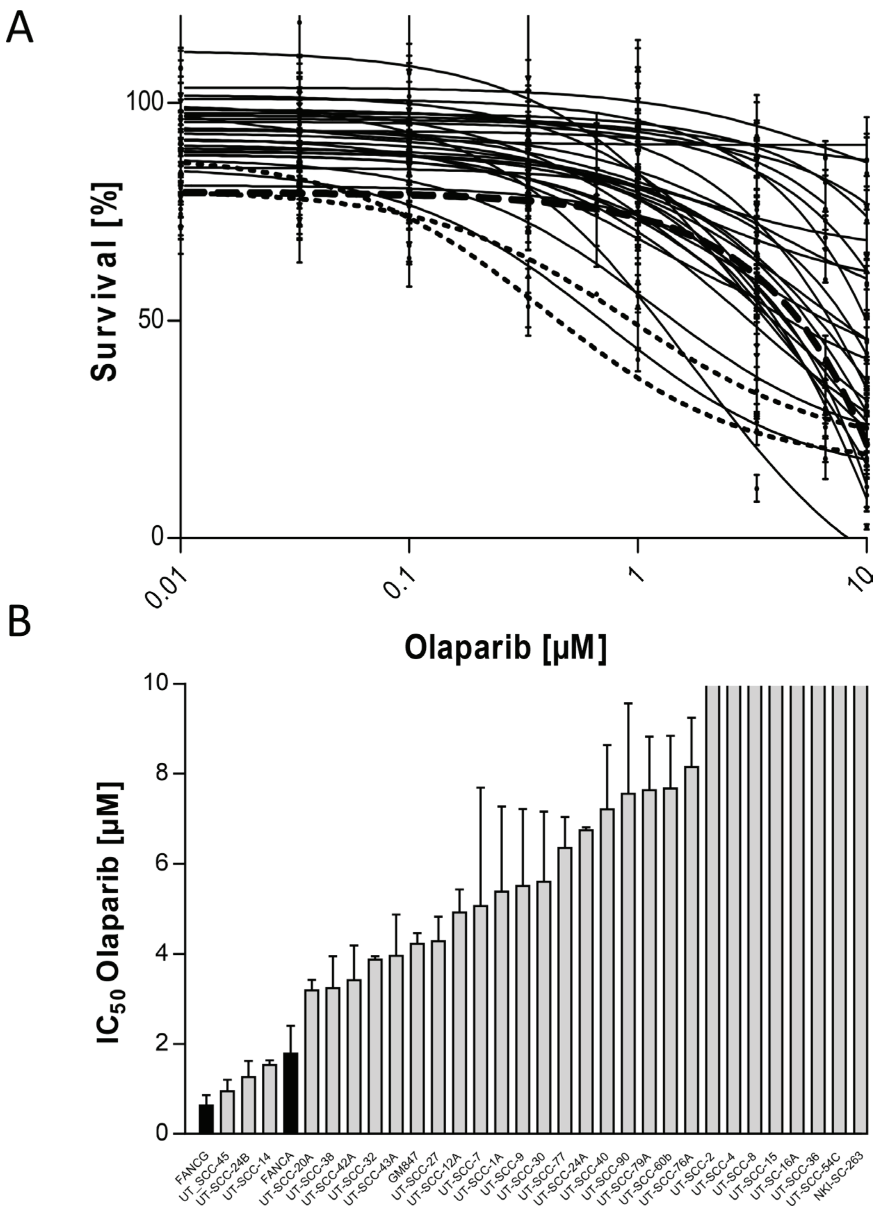

Supplementary Figure 3: Sensitivity of HNSCC cell lines to PARP inhibition by olaparib. (**A**) Olaparib sensitivity in the HNSCC cell line panel as determined by a prolonged growth assay. The average surviving fraction (in %) of three to five independent experiments per cell line is shownas a dose response to the PARP inhibitor olaparib. Olaparib concentrations are log-transformed and non-linear fits on the log-transformed survival data are shown; errors are SEM. The FA control cell lines (EUFA173 and EUFA 636) are depicted by the dotted lines. (**B**) Olaparib IC_{50} values of the individual 29 HNSCC cell lines and FA controls as in A. The average IC_{50} values of each cell line are shown and were determined on the curve fits of the individual experiments; errors are SEM. The highest olaparib concentration that was tested is 10 μM and this value was assigned to olaparib insensitive cell lines in which less than 30% kill was achieved by this dose. FA reference controls are depicted in black.

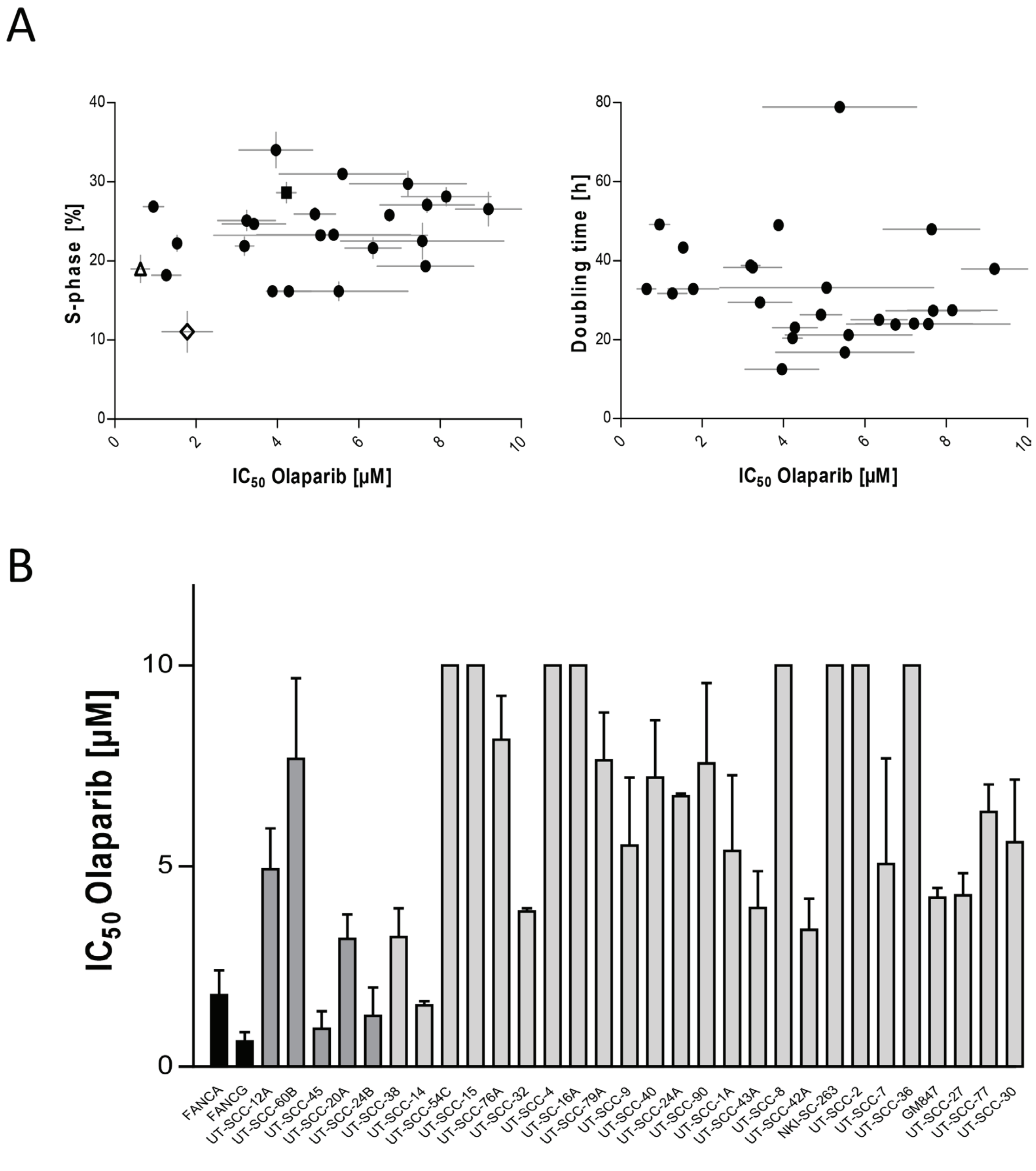

Supplementary Figure 4: Sensitivity of HNSCC cell lines to PARP inhibition by olaparib. (**A**) Comparison of PARP inhibitor olaparib sensitivity to S-phase content in the cell line or to the doubling times as indicated. Olaparib IC_{50} values were compared to S-phase content and doubling times in the HNSCC (filled symbols) and assessed for correlation. The controls (open symbols) and HNSCC cell lines with undetermined IC_{50} values have been excluded for regression and significance analyses. Errors are SEM. (**B**) Olaparib sensitivity of the 29 HNSCC cell lines and controls. The average IC_{50} values of three to five independent experiments are shown for each cell line and were determined on the curve fits of the individual experiments; errors are SEM. HNSCC are ranked according to their MMC sensitivity. The highest olaparib concentration that was tested is 10µM and this value was assigned to olaparib insensitive cell lines.

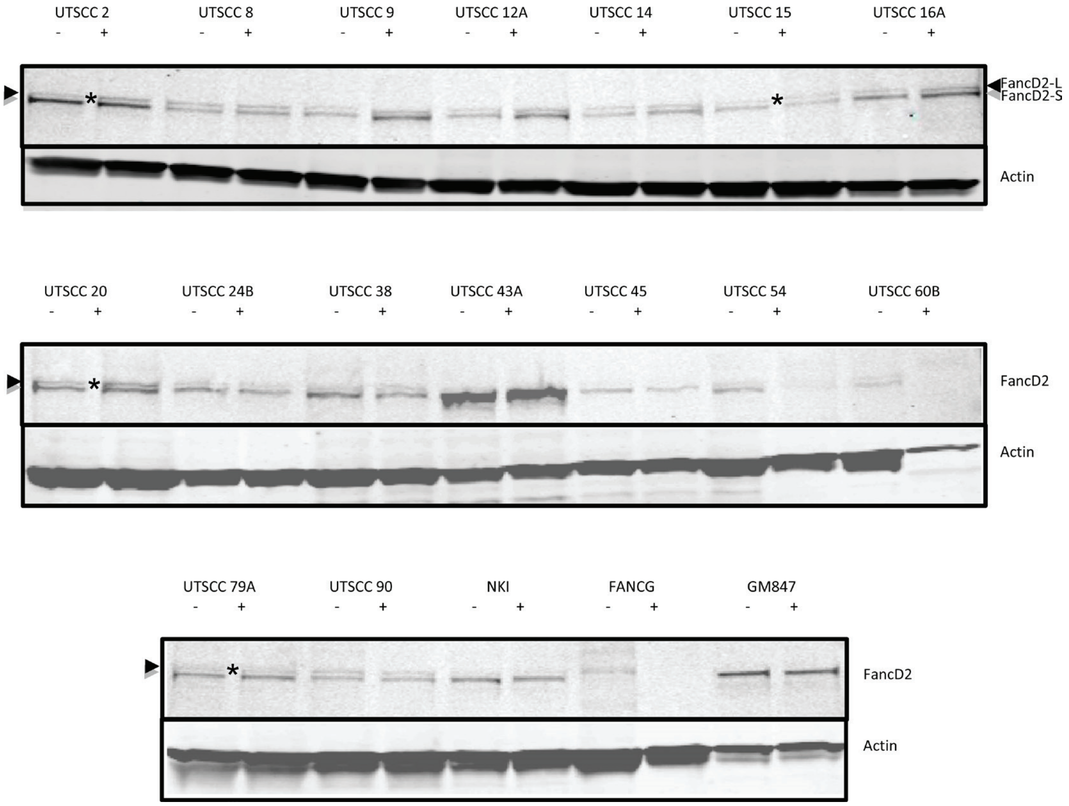

Supplementary Figure 5: FANCD2-monoubiquitylation. Additional representative examples of MMC induced FANCD2-monoubiquitylation in the analyzed HNSCC cell lines. Western images on untreated and MMC treated (36 nM) cell lysates, used for FANCD2-L(ong), indicated by a black arrow head, and FANCD2-S(hort), indicated by a grey arrow head, determinations. Stars (*) indicate positive controls with examples of FANCD2-monoubiquitylation induction by MMC exposure. This is seen by an increase in the intensity in the upper band (FANCD2-L) after MMC treatment. Other HNSCC display signs of FANCD2-monoubiquitylation, even in untreated conditions, and shows FA activation by endogenous sources (replication stress) and is typical for tumor cells. Other HNSCC lack FANCD2-monoubiquitylation (FANCD2-L band). If however sensitive to MMC, one can assume efficient drug activation and this therefore shows the inability to monoubiquitylate FANCD2, such as in the UT-SCC-43A.

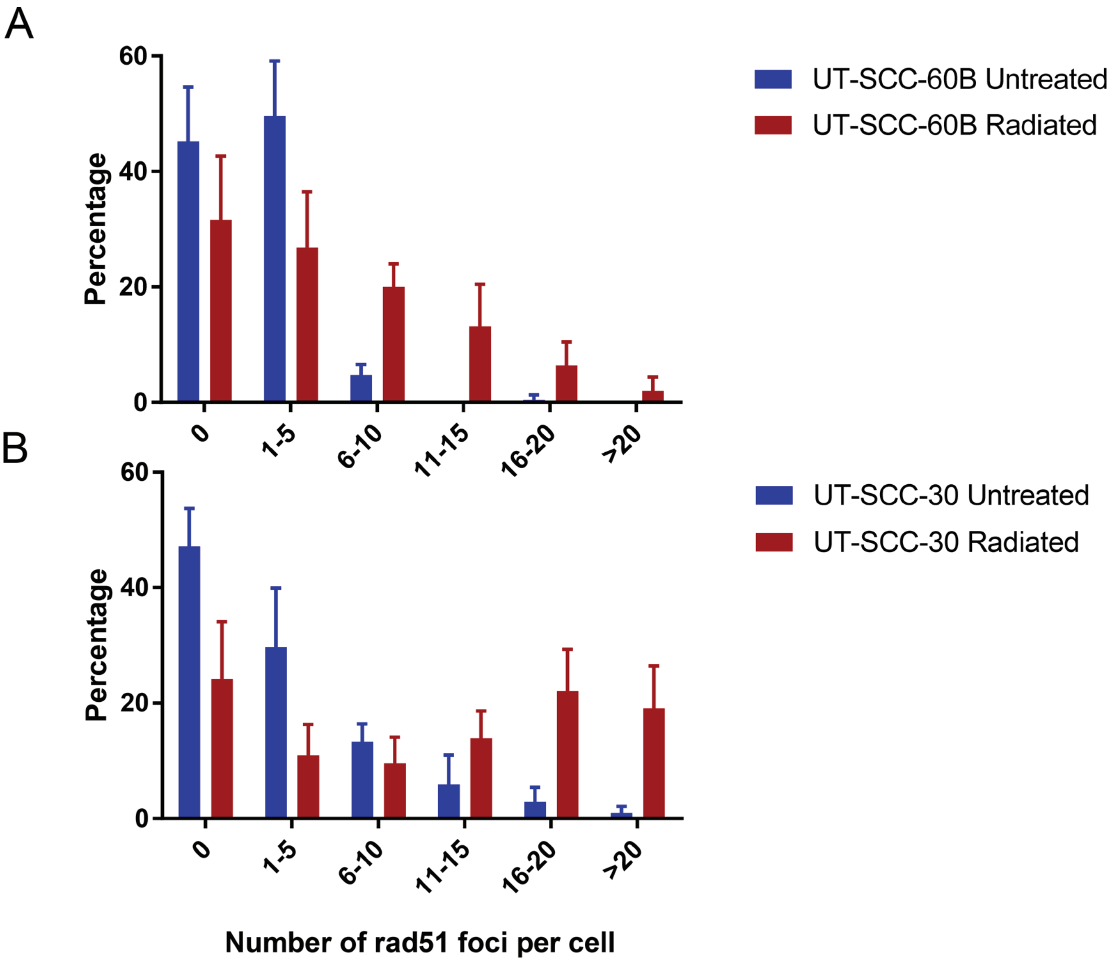

Supplementary Figure 6: Rad51 foci formation after radiation. Radiation-induced RAD51 foci histograms in UT-SCC-60B (**A**) and UT-SCC-30 (**B**). The percentage of cells with rad51foci counts in the indicated bins are shown. In HR-proficient cells radiation (6 Gy) induces the formation of nuclear rad51foci. The data show a pronounced induction of such rad51 foci in the UT-SCC-30 and a greatly dampened response in the UT-SCC-60B. This is consistent with the MMC-hypersensitivity and indicates a compromised FA/HR pathway in the UT-SCC-60B. The average of data of 3-5 independent experiments are shown (error = SD). Cells were seeded in 6-well plates containing sterilized microscope cover slips and irradiated with 6Gy. After 5 hours, cells were fixed with 4% paraformaldehyde, permeablized with 0.15% Triton-X and incubated in 1% BSA. Rad51 labelling was performed by overnight incubation with rabbit anti-RAD51 primary antibody (polyclonal H29, Santa Cruz), followed by a FITC labelled anti-rabbit secondary antibody (Jackson ImmunoResearch) . Images were collected for counting with the Leica TCS SP5 Confocal microscope at 63× or 100× magnification. As shown, UT-SCC-30 which contains a variant in BRCA1, is capable of RAD51 foci formation, indicating an intact repair pathway. This is in accordance with functional BRCA.

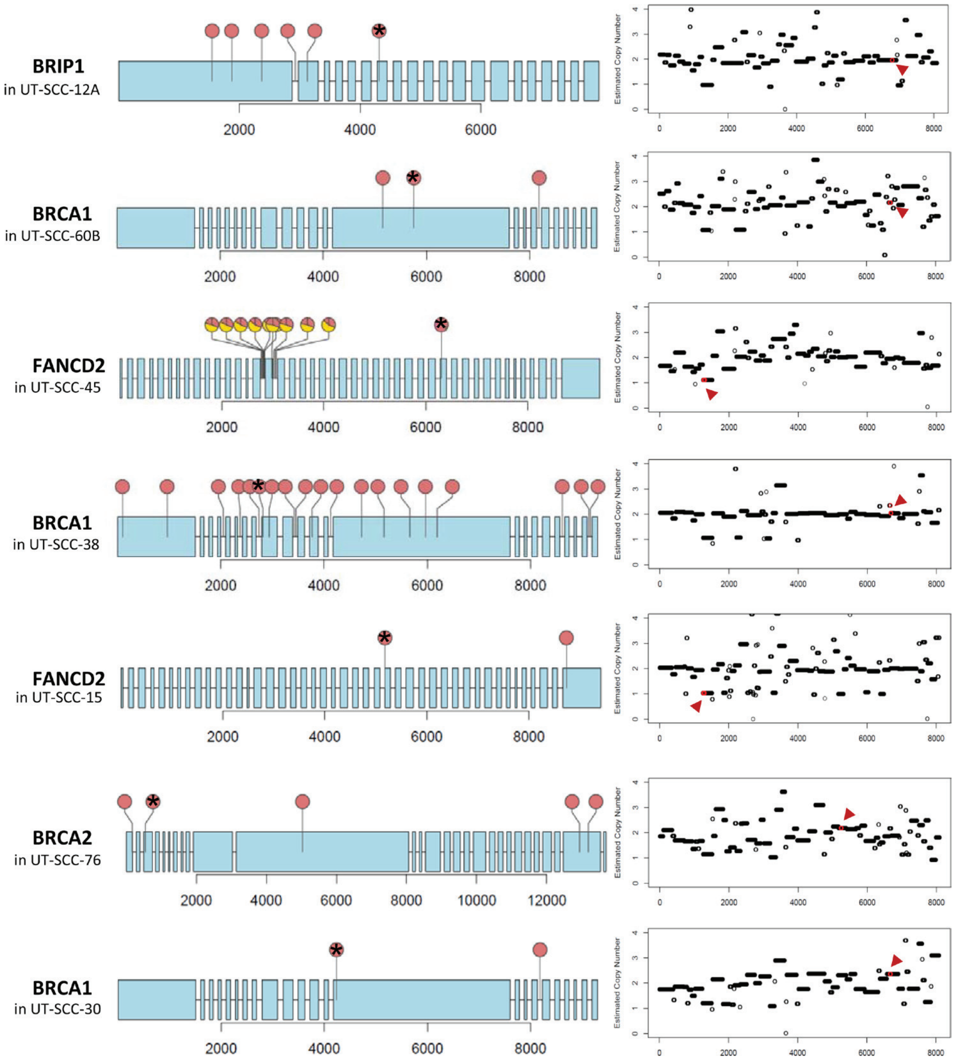

Supplementary Figure 7: Indications for loss of heterozygosity in selected genes. Lolliplot diagrams to the left show the variant zygosity distribution in genes of cell lines that were classified as having a FA/HR variant (marked with *) according to our variant selection protocol. In addition to these variants, all other SNV variants (originally discarded by the variant selection protocol), but with MAF below 60%, are shown. Homozygous variants are shown in red, heterozygous variants in red and yellow. Estimated copy number across all baits in the capture and plotted in chromosomal order (bait No. on the X-axis) are shown to the right for the respective HNSCC line. Red circle and arrow highlight the position of the affected gene as indicated to the left.

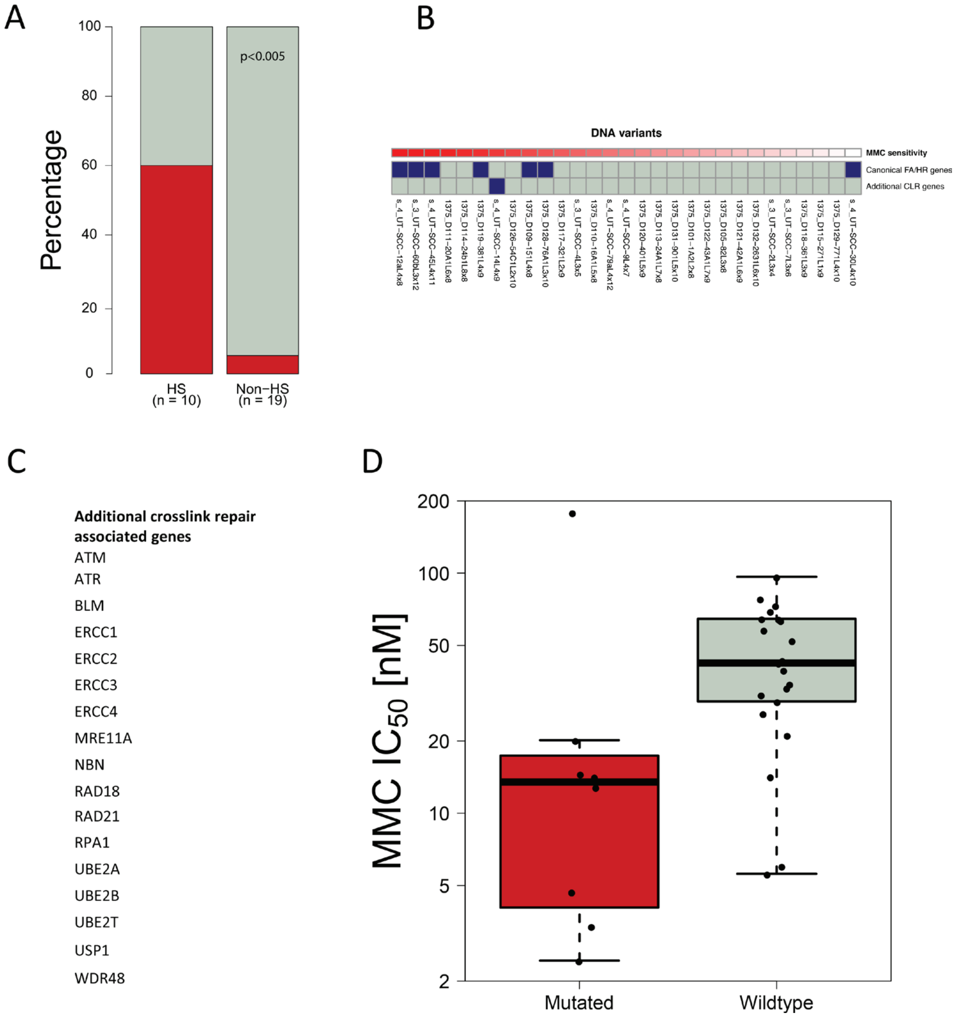

Supplementary Figure 8: FA/HR-variant enrichment in MMC sensitive HNSCC and extension of crosslink repair gene list to peripheral genes for DNA repair defect sequence variant selection. (**A**) FA/HR variants are significantly enriched in the MMC sensitive HNSCC cell lines (HS; top 1/3 most MMC sensitive cell lines) compared to non-HS ($p < 0.005$). (**B**) The variant selection protocol as in Sup-Table 3 was applied to 17 additional genes that act at the periphery of the FA/HR pathway and that could have an impact on crosslink repair (listed under (**C**)). This approach revealed one additional cell line with a potential ATR gene mutation. (**D**) HNSCC cell lines with a variant in any of the 27 FA/HR plus 17 peripheral genes (“mutated”) are significantly more sensitive to MMC (Wilcoxon Rank Sum test: $p < 0.01$; Fisher exact test: $p < 0.005$).

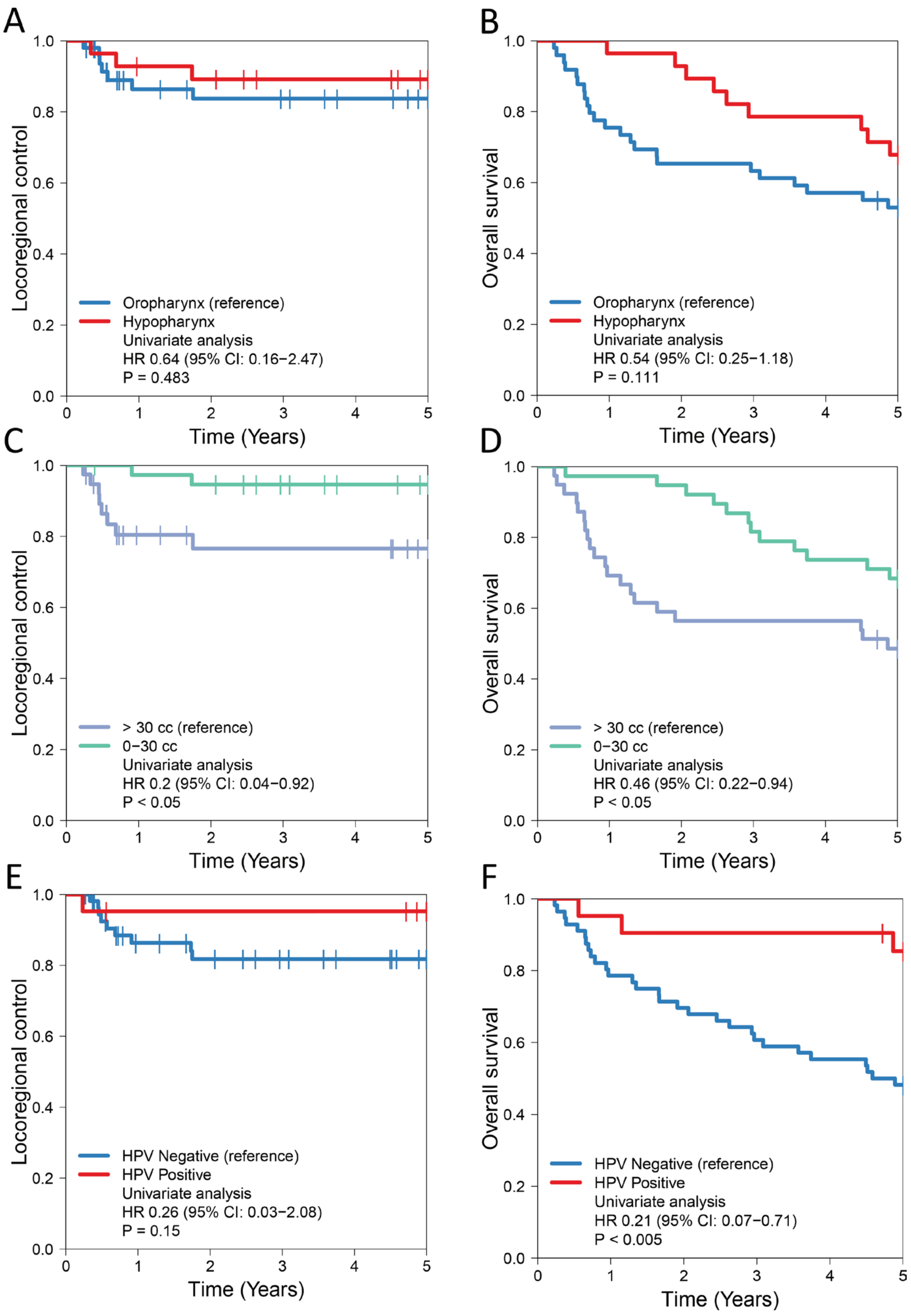

Supplementary Figure 9: Major clinical variables. Locoregional control (**A**, **C**, **E**) and overall survival (**B**, **D**, **F**) in the HNSCC patient cohort showing the influence of (A,B) tumor site (oropharynx vs hypopharynx), (C, D) tumor size (below and above 30 cubic centimeters) and (E, F) HPV status. In-figure: hazard ratios (HR) from univariate Cox models and p-values from the exact log-rank test (see also Supplementary Table 6).

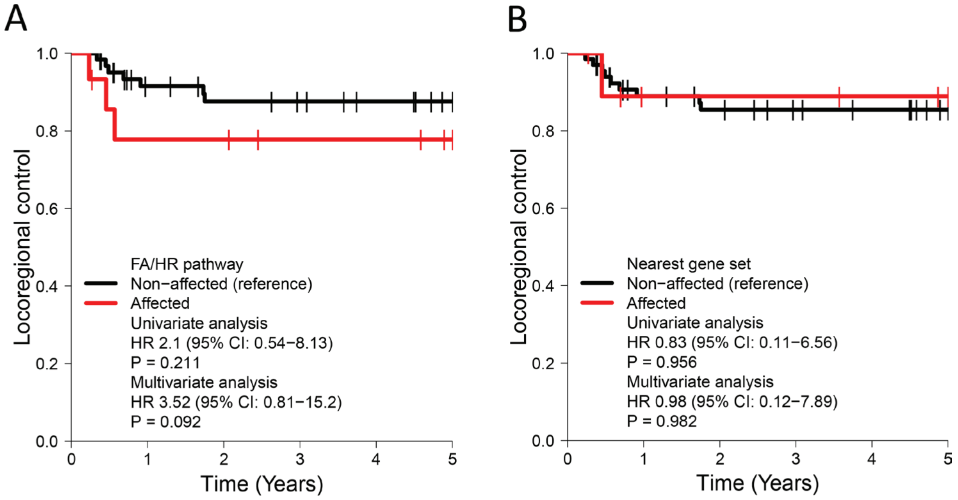

Supplementary Figure 10: Association of functional FA/HR repair defect associated variants with locoregional control in HNSCC patients. FA/HR gene variant selection in the seventy-seven strong chemo-radiated patient cohort with oropharyngeal or hypopharyngeal HNSCC returned 15 patients among the 77 with such tumors. (**A**) Kaplan-Meier graph shows locoregional control in the patients with tumors with and without FA/HR variants as in Figure 4. Data shows a trend to worse prognosis of patients with 'FA/HR affected' tumors. (**B**) Kaplan-Meier graph shows locoregional control in patients with tumors with and without variants in the nearest gene set as in Figure 4D. In-figure: hazard ratios (HR) from Cox proportional hazards models with univariate *p*-values from the exact log-rank test. Multivariate HR and *p*-values from Cox models that include the tested gene set, tumor site, HPV-status and tumor volume are also shown for comparison purposes, however it should be noted that small number of events per variable result in inaccurate parameter estimates and restricts meaningful multivariate analyses. The number of LRC events were 3 for affected and 7 for non-affected.

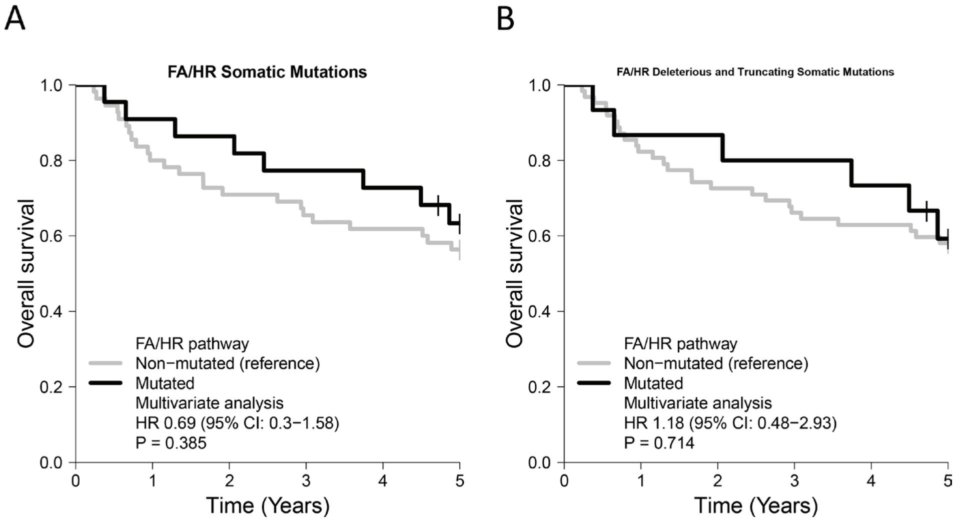

Supplementary Figure 11: Association of somatic FA/HR gene variants with clinical outcome. (**A**) Somatic variant calling in our patient cohort identified 22/77 patient tumors with somatic FA/HR-variants. The somatic variants were not associated with overall survival, showing that common approach of selecting somatic variants would have missed the clinical outcome associations presented in this study. Only variants in exonic and splice site regions were considered. SNP databases were used to remove germline variants, because matched normal samples were unavailable. Variants were considered somatic if they (1) were absent in all three of the 1000 Genomes Project, Exome Sequencing Project or Exome Aggregation Consortium databases, or (2) if they occurred >20 times in the COSMIC database. (**B**) From the somatic FA/HR-variants identified in (A), only those predicted to be deleterious (by SIFT or polyphen) or truncating (frameshift and non-frameshift indels, stop gain and loss variants) were selected. This analysis identified 15/77 patient tumors with deleterious or truncating somatic FA/HR variants. These patient tumors did not have a different outcome than the others. In-figure multivariate hazard ratios (HR) come from multivariate Cox proportional hazard models that include the tested gene set, tumor site, HPV-status and tumor volume.

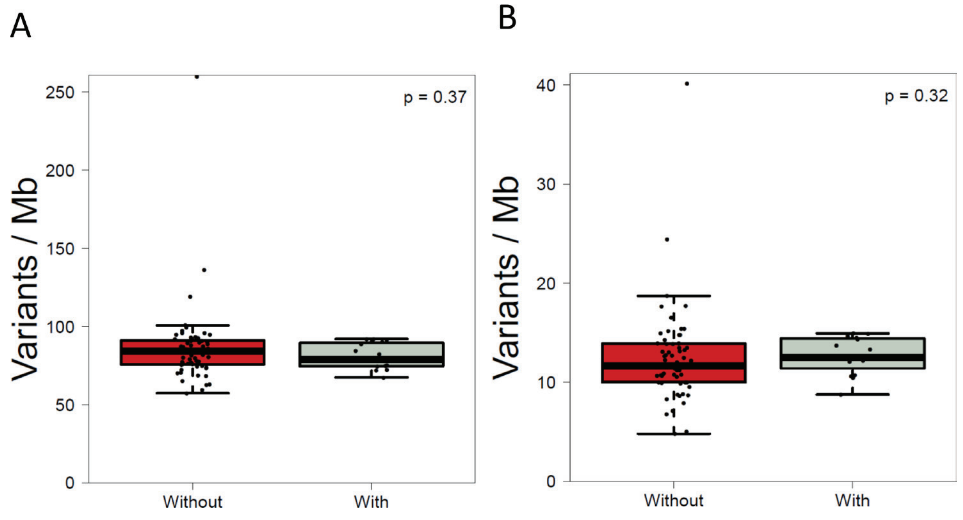

Supplementary Figure 12: Mutational load in tumors with and without a FA/HR-variant. A higher mutational load in tumors (due to inherent mutational processes) can contribute to a higher chance to select sequence variants. Tumors that had FA/HR-variants (i.e were selected after applying the FA/HR variant selection criteria) were compared to those without (Figure 4). (**A**) Non-SNPs sequence variants (synonymous and non-synonymous) representing de novo mutations within all the captured genes (556) were counted. This value was normalized to the individual sample base coverage and is shown as number of variants per sequenced Mb bases for the different tumors. (**B**) Using selection criteria similar to the FA-HR gene variant calling that select for non-synonymous variants and applies a MAF cut off of 2.5% did also not expose any increased variant burden in the sequenced genes in the identified cell lines. Variant load was normalized to the base coverage of the individual samples and is shown as number of variants per sequenced Mb bases. Boxplots are shown with individual tumor sample values as dots.

Supplementary Table 1: Characteristics and sensitivity values of the analysed head and neck cancer cell lines

HNSCC	MMC [nM]			MMC ind. G2 [%]			Olaparib [µM]			AUC		SF2 [Gy]	S-phase content [%]			Doubling time [h]
	IC50	sd	*N*	1 µM	sd	*N*	IC50	sd	*N*		sd		average	sd	*N*	
UT-SCC-1A	43	28,3	4	25	2,7	3	5,4	4,2	5	1.7	0.3	0,38	23	1,0	3	79
UT-SCC-2	65	29,1	3	18	2,6	3	10*		4	1.8	0.2	0,35	22	1,5	3	19
UT-SCC-4	26	15,4	3	29	4,2	3	10*		4	1.7	0.2	0,35	25	1,0	3	30
UT-SCC-7	69	5,5	3	5	2,5	3	5,1	4,6	3	2.0	0.2	0,42	23	1,0	3	33
UT-SCC-8	58	7,3	3	16	2,1	2	10*		3	1.9	0.1	0,37	25	3,8	3	32
UT-SCC-9	33	9,8	3	42	3,3	3	5,5	4,5	7	1.4	0.1	0,25	16	2,0	3	17
UT-SCC-12A	2	0,7	4	53	1,7	3	4,9	1,0	4	2.1	0.1	0,38	26	1,4	3	26
UT-SCC-14	14	8,5	3	17	2,2	3	1,5	0,2	3	1.7	0.3	0,34	22	1,7	3	43
UT-SCC-15	15	6,6	3	22	2,2	3	10*		3	2.1	0.1	0,42	11	1,5	3	49
UT-SCC-16A	29	10,4	5	39	4,3	3	9,2	1,6	4	1.8	0.1	0,33	27	3,7	3	38
UT-SCC-20A	6	2,8	4	49	2,6	3	3,2	0,6	7	2.1	0.2	0,46	22	4,0	12	39
UT-SCC-24A	39	9,4	3	25	3,8	3	6,7	0,1	3	2.6	0.3	0,51	26	1,3	3	24
UT-SCC-24B	6	3,1	5	53	2,0	3	1,3	0,7	4	2.3	0.1	0,43	18	0,0	3	32
UT-SCC-27	78	18,7	3	9	1,7	3	4,3	1,0	3	1.9	0.1	0,37	16	0,0	3	23
UT-SCC-30	179	70,0	4	2	2,1	3	5,6	2,7	3	2.0	0.1	0,39	31	0,6	3	21
UT-SCC-32	21	1,2	3	12	2,6	3	3,9	0,1	3	1.7	0.3	NDT	16	1,0	3	49
UT-SCC-36	73	7,8	3	16	2,1	2	10*		3	2.2	0.2	NDT	25	3,8	3	19
UT-SCC-38	13	3,6	3	24	6,9	3	3,2	1,2	3	2.3	0.3	0,45	25	2,2	3	38
UT-SCC-40	35	4,8	3	32	4,3	3	7,2	2,5	3	2.3	0.2	0,45	30	2,7	3	24
UT-SCC-42A	64	4,4	3	23	1,6	3	3,4	1,9	6	2.1	0.1	0,44	25	1,0	3	29
UT-SCC-43A	52	26,1	4	11	2,6	3	4,0	1,8	4	1.8	0.2	0,34	34	3,9	3	12
UT-SCC-45	5	3,1	4	55	1,9	3	1,0	0,4	3	2.0	0.1	0,37	27	0,6	3	49
UT-SCC-54C	14	5,5	3	22	5,7	3	10*		3	2.3	0.1	0,42	28	2,7	3	52
UT-SCC-60B	3	1,2	3	49	1,2	3	7,7	2,0	3	2.2	0.3	NDT	27	1,7	3	27
UT-SCC-76A	20	11,6	3	17	1,8	3	8,2	2,2	4	2.5	0.2	0,51	28	2,0	3	27
UT-SCC-77	97	17,8	3	22	3,1	3	6,4	1,2	3	2.5	0.2	NDT	22	2,3	3	25
UT-SCC-79A	31	13,1	4	9	3,2	3	7,6	2,1	3	2.4	0.2	NDT	19	1,1	3	48
UT-SCC-90	42	15,2	3	18	2,9	3	7,6	3,5	3	2.2	0.2	NDT	22	3,9	3	24
FANCA	2	0,9	4	44	11,7	2	1,8	1,6	7	NDT	NDT	NDT	11	5,7	5	33
FANCG	2	0,6	4	65	2,8	3	0,6	0,5	5	NDT	NDT	NDT	19	5,9	12	33
GM847	76	34,9	3	11	5,5	3	4,2	0,4	3	NDT	NDT	NDT	29	4,7	13	20
NKI-SC-263	65	30,3	3	54	3,5	3	10*		3	2.51	NDT	0,52	9	1,5	3	24

List of used HNSCC cell lines generated and provided by R. Grenamn and the determined cellular characteristics and response parameters. The FA patient-derived fibroblast cell lines with known mutations in FANCG (EUFA 636)(25) and FANCA (VU0173-F; EUFA 173)(24) were kindly provided by Dr. H. Joenje and served as positive controls, whereas an hTERT transformed human fibroblast cell line (GM847), was included for comparison in a non-tumor derived cell line. The FA gene mutation status in the FANCA and FANCG positive control cell lines was confirmed by PCR and subsequent conventional Sanger sequencing and by capture based sequencing that replicated these results.

Doubling times of all cell lines were assessed by plating different cell numbers into 6-well plates in triplicates and counting live cells after harvesting them by trypsinization at different time points thereafter. Population doubling times (DT) were calculated from the exponential fits on the steepest part of the growth curves. Radiation survival data were kindly provided by R. Grènman.

[*] = IC_{50} was not achieved within the tested dose range; the maximum tested olaparib concentration was assigned to these cell lines. NDT = not determined, sd = standard deviation, AUC = Area under the radiation survival curve, SF2 = surviving fraction at 2Gy, *N* = number of independent experiments. [#] all cell lines were regularly tested for mycoplasma (Janetzko et al, Transfus Med Hemother 2014; 41(1):83-89) twice a month when in culture. Passage number never exceeded 20. Photographs, morphology, cell doubling , PE, characteristics and later DNA-Seq served as authentication method.

Supplementary Table 2: FA/HR gene list

HGNC	ENSEMBL
canonical:	
FANCA	ENSG00000187741
FANCB	ENSG00000181544
FANCC	ENSG00000158169
BRCA2	ENSG00000139618
FANCD2	ENSG00000144554
FANCE	ENSG00000112039
FANCF	ENSG00000183161
FANCG	ENSG00000221829
FANCI	ENSG00000140525
BRIP1	ENSG00000136492
FANCL	ENSG00000115392
FANCM	ENSG00000187790
PALB2	ENSG00000083093
RAD51C	ENSG00000108384
SLX4	ENSG00000188827
RAD51	ENSG00000051180
TP53BP1	ENSG00000067369
BRCA1	ENSG00000012048
FAAP100	ENSG00000185504
RAD50	ENSG00000113522
RAD51B	ENSG00000182185
RAD51D	ENSG00000185379
RAD52	ENSG00000002016
RAD54B	ENSG00000197275
RAD54L	ENSG00000085999
XRCC3	ENSG00000126215
XRCC2	ENSG00000196584

List of canonical FA/HR pathway genes with core activities in the Fanconi anemia and homologous recombination DNA repair pathways that were chosen for the genetic analyses in this study. Canonical FA and HR pathway genes that were available within the sequencing capture set were selected and are listed with their ENSEMBL reference IDs.

Supplementary Table 3: Filtering steps in bio-informatics analysis for variant selection

		**Exclusion criteria:**
Annovar refSeq annotation		
	Region	not annotated as ‘exonic’ or ‘splicing’
	Consequence	Synonymous SNV
Coverage criteria		
	Total reads	<10
	Variant allele containing reads	<4
Healthy blood samples		>1
Minor allele frequency		
	1000 Genomes Project (August 2015, European population)	>0.025[*]
Variant allele fraction		
	Cell lines	<0.80
	Patient samples	<0.80[**]

Variants, single nucleotide variants (SNVs) and indels, were called with VarScan. Only variants that are annotated by RefSeq to be in (ncRNA) exonic or splicing regions were selected. Variant calls were filtered against the following criteria: at least 10 total reads and 4 reads containing the variant allele pass the quality controls, and at least 80% of all reads need to contain the variant allele (variant allele frequency, VAF, of > 0.8). Since assuming loss of gene function to primarily cause the observed DNA repair defects we focused on variants with such a high read fraction, as this is thought to reflect homozygosity. Only rare polymorphisms were retained. This was done by removing variants with a minor allele frequency (MAF) higher than 2.5% in the 1000 Genomes database. Variants that were not present in this database were considered rare or de novo, hence retained. Splicing variants were removed if they were not in COSMIC70. Finally, variants that occurred in two or more of four healthy control subjects that were included in the sequencing analysis were removed. Identified variants were individually confirmed using IGV viewer.

[*]Cut offs were chosen based on HNSCC risk estimates as described in discussion.

[**]After applying sample specific corrections that considered the pathologist’s tumour percentage estimates in the sample as described in Materials and Methods.

Supplementary Table 4: References and characteristics of selected canonical FA/HR gene set variants in HNSCC cell line panel

Cell line	Gene	Protein change	SNP ID	cosmic70	MAF 1000G	CADD	REVEL	SIFT	PolyPhen2	ClinVar	Comments to potential functional effects	PMID
Control FANCA EUFA173	FANCA	R951Q				16.36	0.731		Probably Damaging		described as affecting function and referenced in LOVD; FANCA mutation found in FA patient	17924555
Control FANCG EUFA636	FANCG	T550fs									Frameshift deletion resulting in truncation, FANCG mutation in FA patient	12552564
Control FANCG EUFA636	FANCG	S7F	rs35984312	ID = COSM 753539	0,002	12.71	0.062	Deleterious	B	Benign (Fanconi Anemia)	FANCG mutation found in FA patient	
UT-SCC-12A	BRIP1	G690R				16.23	0.697		Probably Damaging		No report found. The G to R change at the border of the helicase domain could affect the protein function.	
UT-SCC-60B	BRCA1	R841W	rs1800709	ID = COSM 1246204	0,005	5.12	0.355	Deleterious	Possibly Damaging	Benign (hereditary breast cancer)	Referenced in LOVD; Predicted to affect function in evolutionary conservation analysis Likely pathogenic through bayesian analysis considering breast cancer family frequencies 840S is phosphorylation site	12531920 18415037 9585599 8968716
UT-SCC-45	FANCD2	G1043S	rs142238966			15.38	0.169	T	Probably Damaging		Located in DNA Binding region	
UT-SCC-38	BRCA1	M1652I	rs1799967		0,0159	11.83	0.426	T	B	Benign (breast cancer)	Referenced in LOVD: Evolutionary conservation analysis predicts deleteriousness within BRCT domain	18694767 12955716
UT-SCC-15	FANCD2	P834A				19.66	0.414	T	Probably Damaging		Proline to alanine change likely to affect protein structure at DNA binding site	
UT-SCC-76A	BRCA2	Y42C	rs4987046		0,003	4.474	0.089	T	B	Benign (breast cancer)	Alters PALB2 binding site. Variant reported to affect RPA Binding in co-immunoprecipitation assay	12527904
UT-SCC-30	BRCA1	R1347G	rs28897689		0,001	10.36	0.527	Deleterious	B	Benign (breast cancer)	Located at poorly conserved region, predicted to affect function by Grantham score	18415037

PolyPhen-2 (http://genetics.bwh.harvard.edu/pph2/) is a tool that predicts a possible impact of an amino acid change on the protein structure. SIFT (http://sift.jcvi.org/) prediction is based on the degree of conversation of aminoacids in the sequence. CADD (http://cadd.gs.washington.edu/) and REVEL (https://sites.google.com/site/revelgenomics/) are variant effect prediction algorithms that combine the knowledge of a multitude of algorithms such as PolyPhen-2 and SIFT. CADD and REVEL scores above 15 and 0.5, respectively, are considered pathogenic.
T = Tolerated; B = Benign; PMID = reference PubMed ID number. Minor allele frequencies (MAF) from 1000 genome (1000G) are listed.

Supplementary Table 5A: References and characteristics of selected canonical FA/HR gene set variants in the tumor samples of the patients in the study. See Supplementary_Table_5A

Supplementary Table 5B: References and characteristics of selected canonical FA/HR gene set variants in the tumor samples of the patients in the study. See Supplementary_Table_5B

Supplementary Table 6: Statistical values from overall survival analysis

				Univariate Analysis			Multivariate Analysis		
**Variables**		**Patients (*n*)**	**Events (*n*)**	**HR**	**95% CI**	***P*-Value**	**HR**	**95% CI**	***P*-Value**
Tumor site									
	Oropharynx	49	23	1.0 (ref)			1.0 (ref)		
	Hypopharynx	28	9	**0.54**	(0.25–1.18)	*0.1113*	**0.27**	(0.12–0.6)	***0.0014*
Tumor volume									
	> 30 cc	39	20	1.0 (ref)			1.0 (ref)		
	0-30 cc	38	12	**0.46**	(0.22–0.94)	**0.03173*	**0.4**	(0.18–0.87)	**0.0205*
HPV status									
	Negative	56	29	1.0 (ref)			1.0 (ref)		
	Positive	21	3	**0.21**	(0.07–0.71)	***0.00319*	**0.13**	(0.04–0.45)	***0.0012*
Canonical FA/ HR									
	No variant	62	23	1.0 (ref)			1.0 (ref)		
	Variant	15	9	**1.96**	(0.9–4.23)	*0.1035*	**2.59**	(1.11–6.01)	**0.0271*
Nearest genes									
	No variant	67	26	1.0 (ref)			1.0 (ref)		
	Variant	10	6	**1.81**	(0.75–4.41)	*0.2388*	**1.86**	(0.74–4.68)	*0.1889*

Table shows results of the univariate and multivariate analyses. Hazard ratios (HR) and corresponding 95% confidence intervals (95% CI) derived from Cox proportional hazards models are shown for the univariate analysis. The groups were of uneven size, with one group being small, resulting in an inappropriate asymptotic distribution. Therefore exact log-rank test *p*-values which are listed in the table are based on Monte Carlo simulations with 100,000 iterations. Hazard ratios (HR), 95% CI and *p*-values are derived from Cox proportional hazards models in the multivariate analysis. The multivariate model considered tumor site, tumor volume, HPV status, and the genetic variant status as fourth variable. Genetic variant status addressed either the canonical FA/HR variant status or the presence of variants in the nearest genes.

Supplementary Table 7: FA/HR gene mutation and HPV status

A

		HPV status	
		–	+
FA/HR Var	–	43	19
	+	13	2

B

		HPV status	
		–	+
FA/ HR Var	–	22	19
	+	6	2

Table shows the overlap of HPV positivity and FA/HR-variant presence. (**A**) All tumors (n = 77), no significant association between FA/HR variant and HPV status was found. Fishers exact test p = 0.215. (**B**) Only oropharynx tumors (n = 49), no association between FA/HR variant and HPV status was found. Fishers exact test p = 0.4384.

Supplementary Table 8: Matched control gene sets

Canonical FA/HR	Matched nearest
FANCA	CDT1
FANCB	POLA1
FANCC	PTCH1
BRCA2	RFC3
FANCD2	VHL
FANCE	CDKN1A
FANCF	ABCC8
FANCG	APTX
FANCI	POLG
BRIP1	PPM1D
FANCL	PSME4
FANCM	POLE2
PALB2	PLK1
RAD51C	AKAP1
SLX4	ABCA3
RAD51	LTK
TP53BP1	LTK
BRCA1	TOP2A
FAAP100	CSNK1D
RAD50	PPP2CA
RAD51B	HIF1A
RAD51D	LIG3
RAD52	FOXM1
RAD54B	NBN
RAD54L	MUTYH
XRCC3	AKT1
XRCC2	SMARCD3

List of matched genes which are close to the canonical FA/HR pathway genes (Supplementary Table 2). Genes within the > 500 genes strong DNA sequencing capture set that best fulfilled the requirements for matching (nearest) were selected for control analyses.

Supplementary Table 9: RT-PCR primers and kits

Kit/primer	Cat.No:
AllPrep DNA/RNA Mini Kit	Qiagen, Cat. No.80004
QuantiFast SYBR RT-PCR kit	Qiagen, Cat. No.204154
Hs_FANCF_1_SG QuantiTect Primer Assay	Qiagen, QT00241136 (NM_022724)
Hs_GAPDH_2_SG QuantiTect Primer Assay	Qiagen, QT01192646 (NM_002046)
Hs_B2M_1_SG QuantiTect Primer Assay	Qiagen, QT00088935 (NM_004048)

List of primers and kits used for FANCF RT-PCR analysis.
RNA quality was assessed by the Agilent 2100 Bio-analyzer (Agilent Technologies). All RNA samples used for the FANC-F RT-PCR reached maximal RIN 10 values.
