# Supplementary material for: Fanconi anemia and homologous recombination gene variants are associated with functional DNA repair defects in vitro and poor outcome in patients with advanced head and neck squamous cell carcinoma
Source: Oncotarget. 2018 Apr 6;9(26):18198–213. doi: 10.18632/oncotarget.24797 (PMC5915066; doi:10.18632/oncotarget.24797)
Supplement: Supplementary file 2 [file oncotarget-09-18198-s002.docx]

**Supplementary Table 5A: References and characteristics of selected canonical FA/HR gene set variants in the tumor samples of the patients in the study**

| **Pts** | **Gene** | **Protein change** | **SNP ID** | **cosmic70** | **MAF  1000G** | **CADD** | **REVEL** | **SIFT** | **PolyPhen2** | **ClinVar** | **Comments to potential functional effects** | **REF PMID** |
| --- | --- | --- | --- | --- | --- | --- | --- | --- | --- | --- | --- | --- |
| **1** | PALB2 | L337S | rs45494092 |  | 0,0249 | 8.918 | 0.041 | Deleterious | B | Conflicting interpretations | Possibly enriched in familial cutaneous malignant melanoma. Similar frequency in breast cancer than controls. | 24949998  21618343  26283626 |
|  | FANCC | V60I | rs138629441 |  |  | 0.185 | 0.101 | T | B | Likely benign  (Fanconi Anemia) | Unlikely to affect function | 14695169 |
|  | FANCG | R513Q | rs17885240 |  | 0,0129 | 15.01 | 0.016 | T | B | Likely benign (Fanconi Anemia) | Increased frequency in children with AML | 16643430 |
| **2** | PALB2 | T1099R | rs142132127 | ID=COSM1666745 |  | 16 | 0.214 | Deleterious | Damaging | Uncertain significance | Semiconservative AS substitution in conserved 5^th^ WD repeat, region of BRCA2, RAD51 and POLH interaction. Possible enrichment in individuals with breast or ovarian cancer. | 25186627  26315354 |
| **3** | PALB2 | L337S | rs45494092 |  | 0,0249 | 8.918 | 0.041 | Deleterious | B | *as above* | *see above* | *see above* |
| **4** | FANCG | R513Q | rs17885240 |  | 0,0129 | 15.01 | 0.016 | T | B | *as above* | *see above* | *see above* |
| **5** | FANCG | R513Q | rs17885240 |  | 0,0129 | 15.01 | 0.016 | T | B | *as above* | *see above* | *see above* |
| **6** | FANCM | K953N | rs142864437 |  |  | 14.93 | 0.085 | Deleterious | Damaging | Uncertain significance | No reports found |  |
|  | FANCA | A554V |  |  |  | 19.95 | 0.636 | T | Damaging |  | No reports found |  |
| **7** | FANCM | T77A | rs61746895 |  | 0,0129 | 2.724 | 0.041 | T | B | Likely benign (Fanconi Anemia) | Neighboring MPH1 (ERCC4-related helicase) region |  |
| **8** | FANCF | P320L | rs45451294 |  | 0,0119 | 16.83 | 0.096 | T | Possibly  Damaging | Benign (Fanconi Anemia) | No reports found |  |
|  | FANCF | R38H |  |  |  | 23.2 | 0.14 | Deleterious | Damaging |  | No reports found |  |
| **9** | FANCF | P320L | rs45451294 |  | 0,0119 | 16.83 | 0.096 | T | Possibly  Damaging | *as above* | *see above* | *see above* |
| **10** | FANCD2 | N545S | rs145522204 |  | 0,0089 | 0.887 | 0.053 | T | B | Benign | No reports found |  |
| **11** | FANCD2 | R997Q |  |  |  | 15.1 | 0.091 | T | B |  | No reports found |  |
| **12** | FANCC | H256R |  |  |  | 16.51 | 0.058 | T | B |  | Reported in one individual in controls in pancreatic cancer study | 15695377 |
| **13** | RAD51C | G264S | rs147241704 |  |  | 23 | 0.202 |  | B | Uncertain significance | Non-conservative amino acid substitution in conserved region (ATPase domain)  LOVD: hypomorph feature: partial complementation in cells, normal Rad51 foci;  Increased frequency in breast / ovarian Moderate penetrance suggestion in ovarian cancer | 20400964 21990120 |
| **14** | RAD51B | K243R | rs34594234 |  | 0,0089 | 25.4 | 0.186 | T | Damaging |  |  |  |
| **15** | BRCA1 | R841W | rs1800709 | ID=COSM1246204 | 0,005 | 5.12 | 0.355 | Deleterious | Possibly Damaging | Benign (Hereditary Can) | Referenced in LOVD;  Predicted to affect function in evolutionary conservation analysis Likely pathogenic through bayesian analysis considering breast cancer family frequencies neighboring 840S is phosphorylation site | 22753008  21520273  18415037  8968716 |

Abbreviations and listing as in Supplementary Table 4.
