# Supplementary material for: Fanconi anemia and homologous recombination gene variants are associated with functional DNA repair defects in vitro and poor outcome in patients with advanced head and neck squamous cell carcinoma
Source: Oncotarget. 2018 Apr 6;9(26):18198–213. doi: 10.18632/oncotarget.24797 (PMC5915066; doi:10.18632/oncotarget.24797)
Supplement: Supplementary file 3 [file oncotarget-09-18198-s003.docx]

**Supplementary Table 5B: References and characteristics of selected canonical FA/HR gene set variants in the tumor samples of the patients in the study**

| **Pts** | **Gene** | **Protein change** | **Age at diagnosis (years)** | **Primary site** | **HPV-status** | **Tumor volume** | **Smoker** | **Alcohol consumption** |
| --- | --- | --- | --- | --- | --- | --- | --- | --- |
| **1** | PALB2 | L337S | 75 | Hypopharynx | Negative | 0-30 cc | yes | yes |
|  | FANCC | V60I |  |  |  |  |  |  |
|  | FANCG | R513Q |  |  |  |  |  |  |
| **2** | PALB2 | T1099R | 62 | Oropharynx | Positive | 0-30 cc | never | yes |
| **3** | PALB2 | L337S | 60 | Oropharynx | Negative | > 30 cc | yes | yes |
| **4** | FANCG | R513Q | 64 | Hypopharynx | Negative | > 30 cc | yes | former- alcoholic |
| **5** | FANCG | R513Q | 54 | Oropharynx | Negative | 0-30 cc | yes | yes |
| **6** | FANCM | K953N | 55 | Hypopharynx | Negative | 0-30 cc | yes | yes |
|  | FANCA | A554V |  |  |  |  |  |  |
| **7** | FANCM | T77A | 57 | Oropharynx | Positive | > 30 cc | former-smoker | yes |
| **8** | FANCF | P320L | 56 | Oropharynx | Negative | > 30 cc | unknown | unknown |
|  | FANCF | R38H |  |  |  |  |  |  |
| **9** | FANCF | P320L | 59 | Hypopharynx | Negative | 0-30 cc | yes | yes |
| **10** | FANCD2 | N545S | 48 | Oropharynx | Negative | 0-30 cc | yes | yes |
| **11** | FANCD2 | R997Q | 65 | Hypopharynx | Negative | 0-30 cc | former-smoker | never |
| **12** | FANCC | H256R | 64 | Hypopharynx | Negative | 0-30 cc | former-smoker | yes |
| **13** | RAD51C | G264S | 54 | Oropharynx | Negative | > 30 cc | yes | yes |
| **14** | RAD51B | K243R | 73 | Hypopharynx | Negative | 0-30 cc | yes | yes |
| **15** | BRCA1 | R841W | 37 | Oropharynx | Negative | > 30 cc | former-smoker | former -alcoholic |

Continuation of Supplementary Table 5 listing patient characteristics.
